# A conceptual model for advanced/metastatic gastric or gastroesophageal junction cancer: a review of qualitative studies and results from patient interviews

**DOI:** 10.1186/s12885-025-13474-9

**Published:** 2025-01-15

**Authors:** France Ginchereau Sowell, Thom de Milliano, Keri J. S. Brady, Ginamarie Foglia, Medha Sasane, Samira Bensfia, Matthew Reaney

**Affiliations:** 1 Patient Centered Solutions, IQVIA, New York, NY USA; 2 Consulting Services, IQVIA, Amsterdam, Netherlands; 3 Sanofi, Cambridge, MA USA; 4 Sanofi, Bridgewater, NJ USA; 5 Patient Centered Solutions, IQVIA, Reading, UK; 6 Present Address: Shabas Solutions LLC, Fairfax, VA USA; 7 Present Address: Santen Pharmaceutical, Amsterdam, Netherlands

**Keywords:** Advanced/metastatic gastric cancer/Gastroesophageal junction cancer, Conceptual model, Gastric cancer, Gastroesophageal junction cancer, Health-related quality of life, Patient experience data, Patient interviews, Patient-reported outcomes, Qualitative research

## Abstract

**Background:**

Despite approvals of new first-line immunotherapies for advanced/metastatic gastric cancer/gastroesophageal junction cancer (aGC/GEJC), patients’ median survival is around 14 months and their health-related quality of life (HRQoL) is affected by disease-related symptoms and treatment-related side effects. Using a targeted literature review (TLR) and patient interviews, this study identified disease- and treatment-related concepts that are important to patients with aGC/GEJC and their HRQoL.

**Methods:**

A TLR was conducted to identify primary qualitative studies from 2018 to 2021 on patients’ experiences with aGC/GEJC. The results, supplemented with the results of two previously identified studies from 2017, were used to develop a preliminary conceptual disease model of aGC/GEJC and an interview guide. Next, one-to-one concept elicitation interviews were conducted where patients with aGC/GEJC were asked about symptoms, impacts on daily life, experience of care, treatment expectations, and clinical trials. The conceptual model was refined using these patient experience data.

**Results:**

Four studies selected from the TLR and the two previously summarized studies identified 47 symptoms (15 disease-related, 20 treatment-related, 12 disease- and treatment-related) and 35 impacts. Interviews with 20 patients identified 36 symptoms. The 12 most important symptoms (mentioned by ≥ 50% of patients; average disturbance ratings: ≥5, scale: 0 'not disturbing' to 10 'extremely disturbing') were: nausea, fatigue, temperature sensitivity, indigestion, weakness, diarrhea, vomiting, early satiety, swallowing difficulties, taste alterations, abdominal pain, general pain. Symptoms were mostly attributed to systemic treatments (chemotherapy, immunotherapy and targeted therapy), followed by surgery. Thirty-one impacts emerged from the interviews, the most common being emotional disturbances, impacts on daily activities and families, and requiring assistance from caregivers. Patients were mostly positive about their experience of care, willing to enroll in clinical trials, and keen to receive innovative treatments with few side effects. The final conceptual disease model details the symptoms and impacts of aGC/GEJC.

**Conclusions:**

The conceptual model provides valuable data on signs/symptoms and impacts of aGC/GEJC affecting patients’ lives. This can guide the clinical outcome assessment strategy for the development of innovative treatments more comprehensively than input from physicians alone, to ensure treatments improve both patients’ survival and HRQoL. Interviews also help understand patients’ perspectives on clinical trials.

**Supplementary Information:**

The online version contains supplementary material available at 10.1186/s12885-025-13474-9.

## Plain language summary

Gastric cancer is a tumor that starts in the stomach. Gastroesophageal cancer is a type of gastric cancer that starts where the food pipe, or esophagus, connects with the stomach. Together, these two types of cancers are referred to as GC/GEJC. When these cancers spread to other parts of the body, they are said to be advanced or metastatic. This is known as advanced GC/GEJC. Patients with advanced GC/GEJC have many symptoms from the disease. The medicines that patients take to treat or manage their cancer also have side effects. Patients with GC/GEJC often have surgery to remove the tumor in their stomach. Sometimes part or all of the stomach is removed, which can have side effects. The symptoms and side effects from treatments and surgery can have a large impact on patients’ lives. The goal of this study was to understand the experience of patients with advanced GC/GEJC. Researchers wanted to understand which symptoms affect patients the most and whether these symptoms are caused by the disease, the treatment or surgery. They also wanted to find out how advanced GC/GEJC changes patients’ lives, what their family do to help them, and what patients think about the care they receive from physicians and nurses. They were also interested in whether patients are willing to join clinical trials to test new medicines. To answer all these questions, researchers first looked at studies done by other researchers about patients with advanced GC/GEJC. Then, the researchers interviewed patients with advanced GC/GEJC. The researchers interviewed 20 patients in the US who provided their own personal stories about their advanced GC/GEJC. In total, patients mentioned 36 symptoms. Of these, 12 symptoms happened in over half of the patients and were also described as being ‘disturbing’ by patients. These 12 symptoms were: nausea, feeling tired, sensitivity to temperature, indigestion, weakness, diarrhea, vomiting, feeling full early, problems swallowing, changes in taste, stomach pain and general pain. When asked whether symptoms are caused by the disease, the treatment or surgery, patients most often said that symptoms are caused by the treatment, followed by surgery and then the disease. Patients often said that advanced GC/GEJC affected them emotionally. For example, they often felt anxious. They worried about being unable to work and take care of their families and felt scared about their future. Patients also said that advanced GC/GEJC affected how they live their lives, and that they needed daily help. Most patients were happy with the medical care that they were given, were interested in new medicines and were willing to join clinical trials. This information collected directly from the patients will help researchers and physicians to understand better the difficulties of living with advanced GC/GEJC so that these can be considered in future research studies. This information can also be used to identify which symptoms should be targeted by new treatments. It is hoped that these treatments will help increase the length and the quality of life of patients.

**Supplementary Information:**

The online version contains supplementary material available at 10.1186/s12885-025-13474-9.

## Background

Gastric cancer (GC) is an aggressive cancer that ranks as the fifth most frequently diagnosed cancer and the fourth leading cause of cancer-related deaths worldwide [1]. In the USA, it was estimated that around 26,500 new cases of GC were diagnosed in 2023 [2]; approximately 35% of patients with GC present with advanced or metastatic disease at the time of diagnosis [3]. Gastroesophageal junction cancers (GEJCs) constitute approximately 27% of all GCs [4]. Treatments for advanced/metastatic GC, or aGC/GEJC, typically consist of surgery, radiation and/or systemic therapy (chemotherapy, immunotherapy and targeted therapy). However, despite approvals of first-line immunotherapies for aGC/GEJC, patients’ median survival is still poor at around 14 months [5].

Patients with aGC/GEJC experience a high symptom burden that impacts their overall health-related quality of life (HRQoL) [6, 7]. Additionally, patients often suffer from treatment-related signs and symptoms such as abdominal pain, fatigue, nausea, vomiting, and diarrhea [7, 8]. However, a comprehensive understanding of patients’ experiences with aGC/GEJC is missing due to a lack of high quality, in-depth qualitative studies. Research to obtain patient experience data (PED) is therefore needed, which provides valuable information about patients’ signs/symptoms, disease- and treatment-related QoL impacts, unmet needs and priorities.

PED can be used to support clinical trial design, trial endpoint selection and regulatory reviews, including benefit–risk assessments [9], following the US Food and Drug Administration’s (FDA) patient-focused drug development (PFDD) framework [10]. PFDD is now a major focus in the USA, especially since the introduction of the 21st Century Cures Act [11], and it aims to capture and meaningfully incorporate patients’ experiences, perspectives, needs and priorities into drug development and evaluation. PED are also becoming increasingly important in payer decision-making in oncology, especially for accelerated regulatory approvals for cancer treatments aimed at specific patient subpopulations [12].

The aim of this study was to explore the patient experience of aGC/GEJC by conducting a targeted literature review (TLR) of prior qualitative research, followed by in-depth patient interviews. This study explored the signs/symptoms experienced by patients, whether these were associated with the disease, treatment, and/or surgery, and the impact they have on patients and caregivers. Additionally, the study aimed to understand patients’ perspectives on the care they received, their treatment expectations, and their willingness to participate in clinical trials.

## Methods

### Targeted literature review

A TLR was conducted to identify primary qualitative studies on patients’ experiences with aGC/GEJC published between 2018 and 2021. The results of this TLR were supplemented with the results of two studies from 2017 also reporting on the experience of patients with aGC/GEJC: a TLR supplemented with information from patient blogs and insights from oncologists [13] and a qualitative study of patients with aGC/GEJC [14]. The findings were used to develop a preliminary conceptual model covering the experience of patients with aGC/GEJC.

### Patient interviews

The preliminary conceptual model was used to develop a semi-structured interview guide (see Additional file 1, Interview guide summary) and qualitative telephone interviews were conducted with patients with aGC/GEJC to collect information on the patient experience and finalize the conceptual model. The study protocol and the informed consent form were also developed using the findings from the TLR. All the templates of the study materials were approved by the Institutional Review Board (IRB), Western Institutional Review Board (WIRB^®^, now known as WIRB-Copernicus Group: WCG^®^ IRB; Review number: 44798204).

#### Recruitment

Patients from the USA with aGC/GEJC (see Supplementary Table 1 for the full eligibility criteria) were recruited between February and August 2021 using a patient advocacy group (Debbie’s Dream Foundation: Curing Stomach Cancer, Fort Lauderdale, FL, USA) and two recruitment companies (Rare Patient Voice and Global Perspectives). Patients were recruited via social media posts, membership newsletters and emails, and completed an online form to consent to eligibility screening. Once screened, eligible patients provided informed consent for the study online and were made aware that the study was being conducted by IQVIA on behalf of a pharmaceutical company (the name of the pharmaceutical company was not disclosed). Patients’ treating physicians were required to complete a confirmation of diagnosis form prior to the interviews.

#### Interview process

The study involved individual, one-to-one, semi-structured telephone interviews that were approximately 75 to 90 min in length. The interviews were audio-recorded and verbatim transcripts from each interview were developed and used as source data for the analysis. Patients were first asked to spontaneously describe the signs, symptoms, and impacts they experienced; afterward, they were probed using a list of signs, symptoms, and impact concepts included in the preliminary conceptual model. Patients were asked to rate the disturbance of signs/symptoms that they experienced on a scale from 0 to 10, where 0 is ‘not disturbing at all’ and 10 is ‘extremely disturbing’. Signs/symptoms mentioned by ≥ 50% of patients with an average disturbance rating ≥ 5 were considered most important.

#### Concept saturation for signs/symptoms and impacts

Throughout the study, researchers assessed saturation of concepts, e.g., the point at which additional patient interviews do not contribute unique concepts or new information [15, 16]. Saturation was assessed for signs/symptoms and impacts throughout the study to ensure adequate sample size. To assess saturation, interview transcripts were organized chronologically and grouped in sets (or waves), each containing five transcripts. Concepts mentioned by patients during a set of five interviews were compared with the concepts mentioned in the previous sets. If new concepts were mentioned by a patient in a set of interviews, no concept saturation was achieved. This comparison was repeated for each of the four sets of five interviews (*N* = 20), comparing each set of interviews with all the previous sets, and the point at which saturation was achieved was identified. A sample size of 20 patients was anticipated to be sufficient to reach saturation of concepts [17].

#### Coding of transcripts

To ensure consistency in the data capture and analysis, the de-identified transcripts of patient interviews were coded using a qualitative research software (Atlas.ti). Two experienced researchers were independently involved in the coding process. Before coding began, the study team designed a codebook that captured all signs, symptoms, and impacts identified in the preliminary conceptual model. Each transcript was coded according to the study codebook, which was updated throughout the study as new concepts emerged from the interviews. The coding team conducted regular reviews to ensure the correct updating of the codes. A coding log was created to allow the two coders to assess the limitations of the codebook and suggest improvements. Regular alignment meetings were held between a senior researcher and the two coders to assess the adequacy of the codebook, as well as resolve any discrepancies and elements that were redundant. Intercoder agreement (predefined as Krippendorff’s C-Alpha Binary > 0.7) was reached by transcript two and all remaining transcripts were coded independently. During disturbance ratings of signs/symptoms, where patients reported a rating between two numbers (e.g., between 6 and 7), the highest disturbance rating mentioned was coded (therefore a “6 or 7” became a 7). Once coding was completed, data from Atlas.ti were exported to Excel for analysis and for quality assurance purposes.

#### Refinement of the conceptual model

The preliminary conceptual model developed based on the TLR was updated based on the data obtained from patient interviews and the final conceptual model was developed.

## Results

### Targeted literature review

The TLR identified four studies and the results from these were combined with data from the two earlier studies previously summarized by the researchers. An overview of these six studies is reported in Supplementary Table 2.

From these 6 studies, numerous disease-related (*n* = 15) and treatment-related (*n* = 20) signs/symptoms were identified, as well as other signs/symptoms (*n* = 9), which were reported as being related to both disease and treatment (Supplementary Table 3). A wide variety of impacts of aGC/GEJC and/or treatment (*n* = 35) were reported (Supplementary Table 4). These findings were used to develop a preliminary conceptual model. Given the large number of concepts identified, the preliminary conceptual model included only the signs/symptoms and impacts with an estimated prevalence of ≥ 50% (i.e., at least one literature source reported a prevalence of ≥ 50% spontaneous mentions by patients; *n* = 15 symptoms and *n* = 11 impacts; Supplementary Fig. 1).

### Patient interviews

#### Patient characteristics

Patient characteristics are reported in Table 1. In total, 20 patients with aGC/GEJC were interviewed. The majority of patients (*n* = 13, 65%) were women and nearly all patients (*n* = 19, 95%) were White. The mean time from the original diagnosis was 3.6 years (range: 0.3–12 years). Most patients (*n* = 12, 60%) had previously undergone surgery, with some of these patients undergoing full gastrectomy along with removal of multiple organs due to metastasis. Patients had a diverse medication history: 85% had previously received chemotherapy (*n* = 17), 50% targeted therapy (*n* = 10) and 40% radiation therapy (*n* = 8). At the time of the interviews, 50% of the patients (*n* = 10) were receiving chemotherapy and/or targeted therapies, while only 2 patients (10%) were receiving experimental agents. No patients were currently receiving radiation therapy and 30% of patients had previously participated in a clinical trial.

**Table 1:** Patient and clinical characteristics

Characteristic (*n* = 20)	Value
**Age**,** years**,** mean (min–max)**	58 (33–75)
**Sex**	
Women Men	13 (65) 7 (35)
**Race/ethnicity**	
White Asian Black/African American Native American Hispanic/Mexican	19 (95) 0 (0) 0 (0) 0 (0) 1 (5)
**Level of education**	
High school/college Bachelor’s degree Higher level education (e.g., Masters, PhD)	5 (25) 11 (55) 4 (20)
**Employment status**	
Actively employed/working Unemployed and not looking for work at the moment Retired	7 (35) 5 (25) 8 (40)
**Disease status** ^**a**^	
aGC aGEJC	12 (55) 8 (45)
**Mean time since original diagnosis**,** years (range)**	3.5 (0.3–12)
**Surgery**	
Underwent surgery for aGC/GEJC Did not undergo surgery for aGC/GEJC	12 (60) 8 (40)
**Previous treatments received for aGC/GEJC** ^**b, c**^	
Chemotherapy Radiation therapy Targeted therapies (e.g., nivolumab, trastuzumab, pembrolizumab, ramucirumab, or trastuzumab deruxtecan)	17 (85) 8 (40) 10 (50)
**Current treatment received for aGC/GEJC** ^**b, c**^	
Time on current treatment, months, mean (range)^d^ Chemotherapy Radiation therapy Targeted therapies (e.g., nivolumab, trastuzumab, pembrolizumab, ramucirumab, or trastuzumab deruxtecan) Experimental agent No current treatment	11 (1–65) 10 (50) 0 (0) 10 (50) 2 (10) 5 (25)

All data are presented as number (%) unless otherwise indicated

^a^Disease status based upon confirmation of diagnosis forms

^b^Please note that patients may receive multiple treatments at once (e.g., radiation + chemotherapy or chemotherapy + targeted therapies). Treatments based on patient self-reported therapies in the screening document and during interviews

^c^Information on past and current treatments was derived from the detailed list of past and current treatments reported by patients during the interviews

^d^Information on time on current treatment was calculated using the data available on the patient screeners

aGC/GEJC, advanced/metastatic gastric cancer/gastroesophageal junction cancer

### Concept elicitation

#### Concept saturation of signs/symptoms and impacts

A total of 36 signs/symptoms were reported by patients (Table 2). Most signs/symptoms (81%) were initially reported during the first wave of five interviews. During the second wave of interviews, three new signs/symptoms (8%) emerged. During the third wave of interviews, four new signs/symptoms (11%) emerged. By the fourth wave of interviews, no new signs/symptoms were reported by patients, indicating that concept saturation of signs/symptoms had been reached after 15 interviews.

**Table 2:** Frequency of aGC/GEJC signs/symptoms per attribution and average disturbance rating

Symptom	Total patients mentioning, *n* (*n* = 20)	Spontaneous mentions, *n* (%)^a^	Attribution to disease/ treatment/surgery^b^ *n* (%)			Total patients reporting disturbance ratings, *n* (%)	Mean disturbance rating^c^ (SD)
			Disease (*n* = 20)	Treatment (*n* = 20)	Surgery (*n* = 12)		
**Nausea**	20	18 (90)	4 (20)	20 (100)	5 (25)	14 (70)	**6.9 (3.4)**
**Weight loss**	20	16 (80)	8 (40)	12 (60)	4 (20)	13 (65)	4.4 (4.5)
**Fatigue**	20	20 (100)	9 (45)	20 (100)	4 (20)	18 (90)	**8.1 (2.0)**
**Neuropathy**	18	9 (50)	0 (0)	16 (89)	0 (0)	15 (83)	4.9 (3.0)
**Sensitivity to temperature**	17	9 (53)	2 (12)	16 (94)	0 (0)	12 (71)	**6.8 (2.8)**
**Indigestion**	17	13 (76)	7 (41)	1 (6)	7 (41)	11 (65)	**6.6 (3.4)**
**Constipation**	15	6 (40)	2 (13)	8 (53)	1 (7)	11 (73)	4.9 (3.1)
**Diarrhea**	15	8 (53)	1 (7)	11 (73)	2 (13)	9 (60)	**5.7 (3.2)**
**Hair loss**	15	9 (60)	0 (0)	13 (87)	0 (0)	10 (67)	4.5 (3.5)
**Early satiety**	14	4 (29)	4 (29)	5 (36)	5 (36)	9 (64)	**6.4 (3.2)**
**Flatulence**	14	3 (21)	3 (21)	5 (36)	4 (29)	10 (71)	4.5 (2.5)
**Taste alterations**	14	4 (29)	0 (0)	11 (79)	0 (0)	10 (71)	**7.3 (2.4)**
**Difficulty in swallowing**	14	12 (86)	6 (43)	6 (43)	4 (29)	7 (50)	**8.7 (1.9)**
**Vomiting**	14	10 (71)	2 (14)	9 (64)	2 (14)	10 (71)	**7.9 (3.2)**
**Abdominal pain**	13	8 (62)	7 (54)	1 (8)	5 (38)	10 (77)	**7.6 (2.5)**
**Weakness**	13	12 (92)	5 (38)	10 (77)	3 (23)	10 (77)	**7.1 (2.4)**
**Loss of appetite**	12	7 (58)	5 (42)	9 (75)	4 (33)	11 (92)	4.9 (4.3)
**Other pain**	12	8 (67)	4 (33)	5 (42)	3 (25)	5 (42)	**6.2 (3.6)**
**Mouth sores**	9	5 (56)	0 (0)	6 (67)	0 (0)	5 (56)	**7.4 (2.6)**
**Skin problems**	9	6 (67)	0 (0)	8 (89)	0 (0)	5 (56)	**5.2 (3.6)**
**Bloating**	8	3 (38)	3 (38)	3 (38)	1 (13)	7 (88)	**6.3 (1.4)**
**Headache**	7	2 (29)	1 (14)	3 (43)	0 (0)	4 (57)	**5.8 (1.9)**
**Excessive sweating**	6	1 (17)	0 (0)	3 (50)	1 (17)	5 (83)	**7.0 (1.9)**
**Breathing problems**	6	3 (50)	1 (17)	2 (33)	0 (0)	2 (33)	**9.0 (1.4)**
**Hearing impairment**	5	2 (40)	0 (0)	4 (80)	0 (0)	3 (60)	4.0 (2.6)
**Swelling**	5	3 (60)	0 (0)	3 (60)	1 (20)	2 (40)	**7.0 (2.8)**
**Eye problems**	5	1 (20)	0 (0)	4 (80)	0 (0)	3 (60)	3.3 (1.2)
**Bleeding**	5	5 (100)	2 (40)	1 (20)	0 (0)	1 (20)	4.0 (NA)
**Hiccups**	4	0 (0)	0 (0)	0 (0)	2 (50)	0 (0)	NP (NA)
**Back pain**	4	4 (100)	2 (50)	0 (0)	0 (0)	0 (0)	NP (NA)
**Spasms**	4	4 (100)	0 (0)	1 (25)	0 (0)	0 (0)	NP (NA)
**Bone pain**	4	4 (100)	1 (25)	3 (75)	0 (0)	1 (25)	**9.0 (NA)**
**Sensitivity to odor**	3	3 (100)	0 (0)	2 (67)	0 (0)	1 (33)	**9.0 (NA)**
**Gum problems**	2	2 (100)	0 (0)	2 (100)	0 (0)	1 (50)	**6.0 (NA)**
**Itching**	1	1 (100)	0 (0)	0 (0)	0 (0)	0 (0)	NP (NA)
**Cough**	1	1 (100)	0 (0)	0 (0)	0 (0)	0 (0)	NP (NA)

^a^Patients who did not mention the concept spontaneously were asked directly about the symptom

^b^Not all patients expressed if they related the symptom to disease, treatment, and/or surgery; some patients attributed symptoms to all three factors: disease, treatment, and surgery

^c^Disturbance ratings ≥ 5 are indicated in bold. Disturbance ratings were assessed on a 0–10 scale, where 0 = not disturbed at all and 10 = extremely disturbed. Disturbance ratings were based on the number of patients who provided a rating, which does not always coincide with the number of patients who endorsed the symptom. Some patients provided qualitative descriptions but did not provide a quantitative response

aGC/GEJC, advanced/metastatic gastric cancer/gastroesophageal junction cancer; NA, not applicable; NP, not provided; SD, standard deviation

A total of 31 impacts were reported by patients. Most impacts (90%) were initially reported during the first wave of five interviews. During the second wave of interviews, only two new impacts (6%) emerged, and during the third wave only one new impact (3%) emerged. By the fourth wave of interviews, no new impacts were reported by patients and concept saturation of impacts was reached.

#### Signs/symptoms

Of the 36 unique signs/symptoms that emerged during the interviews, 18 were mentioned by ≥ 50% of the patients. Signs/symptoms described by patients are shown in Table 2 and all patient quotations are reported in Supplementary Table 5.

**Fig. 1:**
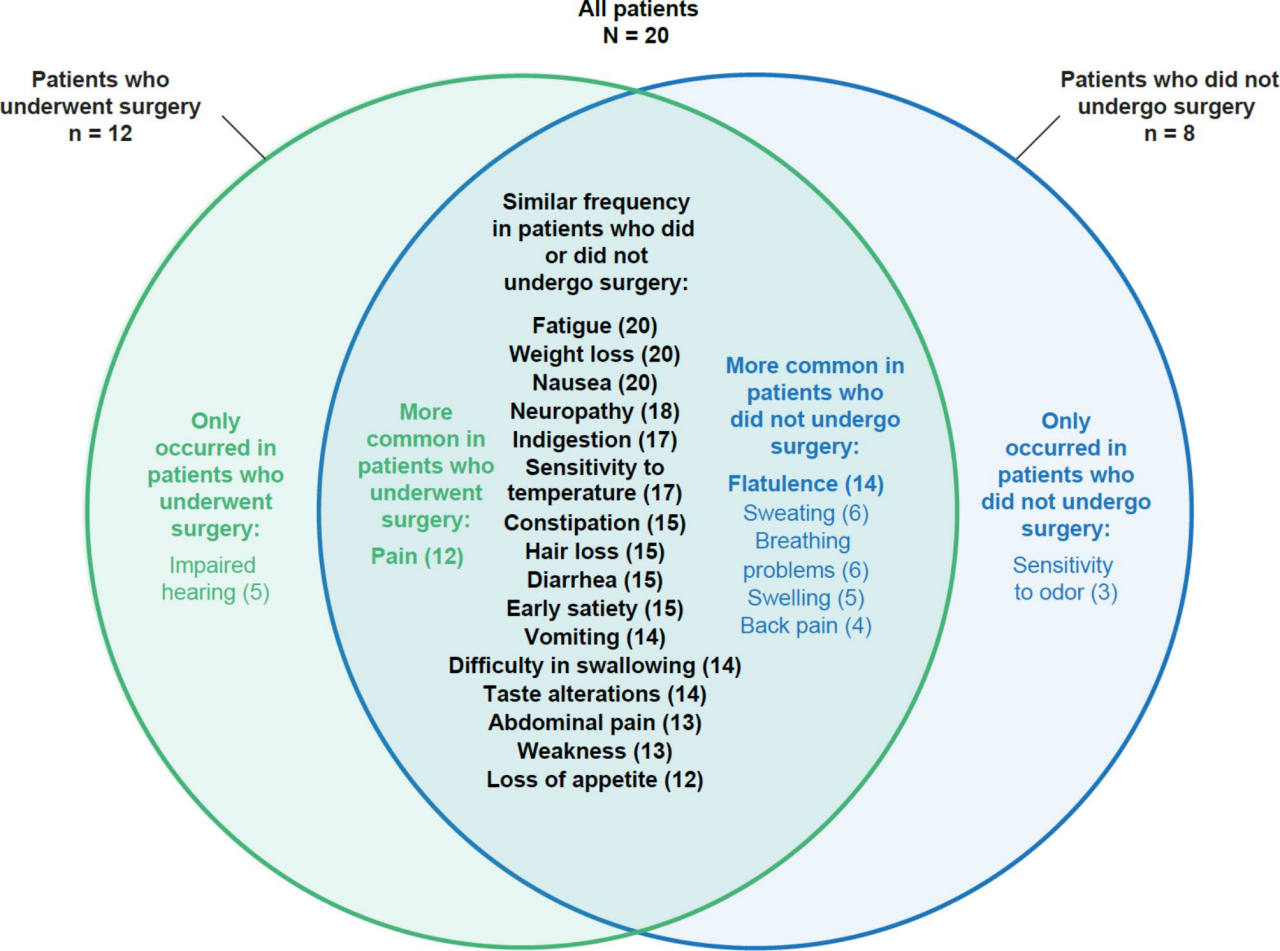
Venn diagram of the frequency of signs/symptoms in patients who had surgery compared with patients who did not have surgery The most important signs/symptoms (frequency ≥ 50% and an average disturbance rating of ≥ 5) are indicated in bold. Numbers in brackets are the number of patients who reported experiencing each symptom

There were some differences in signs/symptoms between patients who had undergone surgery (*n* = 12/20) and patients who had not undergone surgery (*n* = 8/20) (Fig. 1). Impaired hearing (42% vs. 0%) and general pain (75% vs. 38%) were more common in patients who had versus had not undergone surgery. Conversely, flatulence (88% vs. 58%), back pain (38% vs. 8%), sweating (50% vs. 17%), breathing problems (50% vs. 17%), sensitivity to odor (38% vs. 0%), and swelling (50% vs. 8%) were more common in patients who had not undergone surgery versus those who had.

#### Signs/symptoms that were most important to patients

**Fig. 2:**
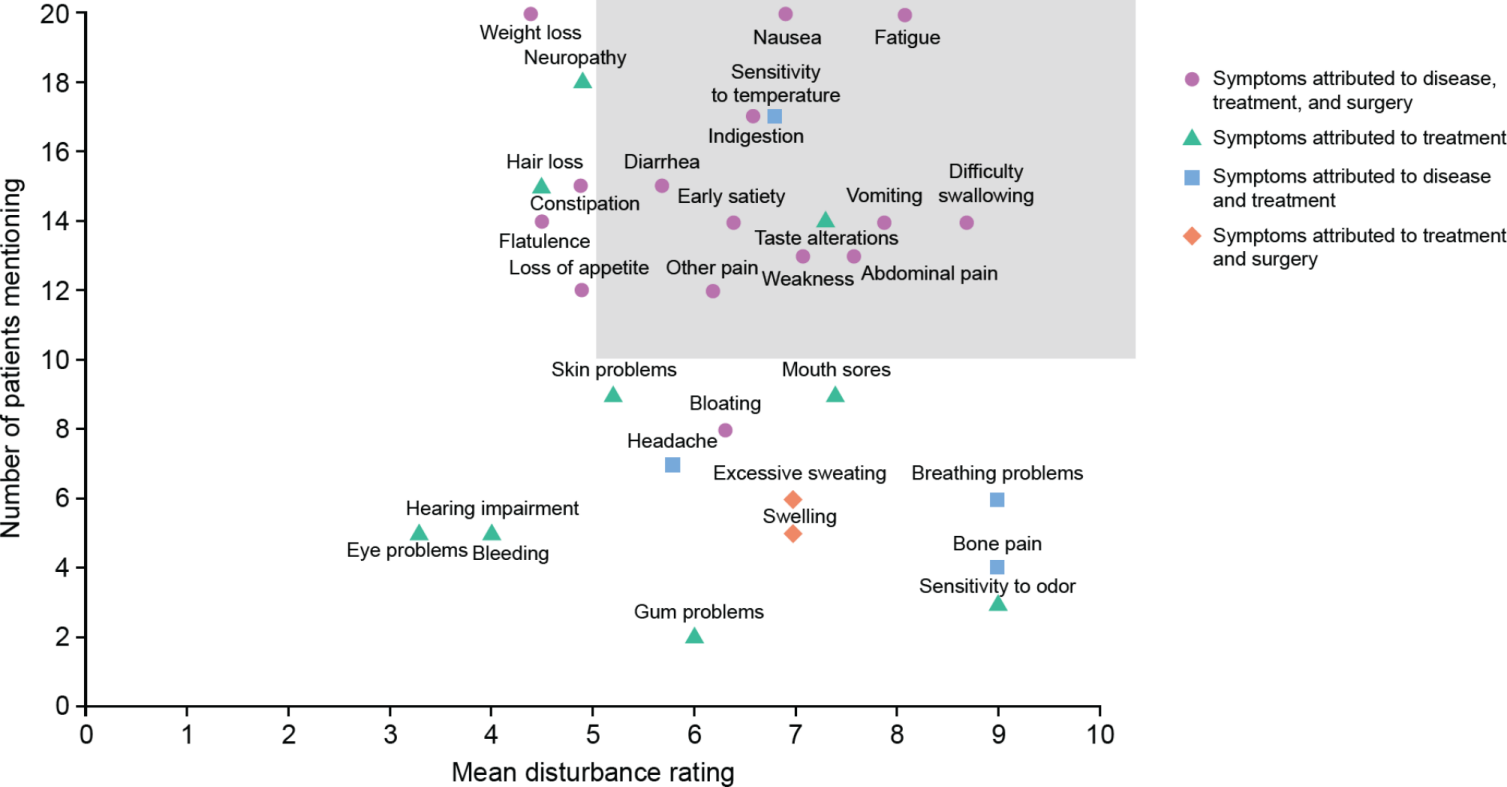
Frequency and disturbance rating of sign/symptoms Signs/symptoms present in ≥ 50% of the patients were defined as being the most important

The following 12 signs/symptoms were considered most important by patients (mentioned by ≥ 50% of patients and with a score ≥ 5 out of a rating scale of 0 = not disturbing at all to 10 = extremely disturbing): nausea, fatigue, sensitivity to temperature, indigestion, weakness, diarrhea, vomiting, early satiety, difficulties in swallowing, taste alteration, abdominal pain, and general pain (Fig. 2).

### Nausea

All patients (20/20, 100%) reported nausea and attributed it to treatment and a small proportion of patients also attributed nausea to the disease (4/20, 20%) and to surgery (5/20, 25%; 5/12, 42% of the patients who had also undergone surgery). Nausea ranged from mild to extremely severe and was typically reported after treatment. Some patients described that nausea was constant throughout the day but gradually subsided after the cessation of treatment. Nausea was associated with early satiety, sensitivity to odor, acid reflux, and taste alterations. The following quotation offers an example of how one patient reported nausea: *“I had significant nausea and vomiting and even from the very first cycle. I felt bad anyway*,* and then the chemo made me feel worse.* (…) *I have significant reflux disease. When I have an episode of that*,* it results in nausea and often vomiting.”*

### Fatigue

All patients (20/20, 100%) reported fatigue and attributed it to treatment. In addition, a considerable proportion of patients (9/20, 45%) also attributed fatigue to the disease and a smaller proportion to surgery (4/20, 20%; 4/12, 33% of the patients who had also undergone surgery). Most patients felt constantly fatigued and described the impact of fatigue on their activities of daily living. Similarly to what patients reported with nausea, fatigue was reported to increase during treatment. Poor food intake caused by nausea also contributed to fatigue, as did weight loss, anemia, vomiting, and early satiety. Fatigue was described as different from usual tiredness: one patient referred to it as a *“strange feeling.”* Another patient described how fatigue felt: “*At its worse*,* I just don’t get out of bed for 30 plus hours. I just sleep most of the time and that’s about it at its worst.”*

### Sensitivity to temperature

Most patients (17/20, 85%) mentioned sensitivity to temperature, and most (16/17, 94%) attributed this to chemotherapy. Patients reported being sensitive to hot or cold surfaces and to hot or cold food or beverages. Signs/symptoms of temperature sensitivity subsided in most patients after discontinuation of chemotherapy. One patient reported: *“The cold sensitivity when it was happening was the worst*,* I think.”*

### Indigestion

Most patients (17/20, 85%) reported indigestion and issues with regurgitation and this was attributed to surgery by 41% (7/17) of patients (7/12, 58% of the patients who had also undergone surgery) and to the disease by 41% (7/17) of the patients. Only one patient (1/17, 6%) attributed indigestion to treatment. Patients often referred to indigestion as acid reflux or a *“burning sensation when eating”*. Due to indigestion, patients were unable to eat and reported weight loss. In addition, acid reflux induced vomiting in some patients. One patient described their experience with acid reflux as follows: *“I have a lot of acid reflux. Well*,* it’s not acid reflux. I have to take that back. I have bile reflux because I don’t have a stomach*,* so what the gastroenterologist told me is happening is that the bile backs up into my esophagus*,* so I have a medication that pushes that back down and helps tremendously to take care of that (…) (It’s related) to the surgery (…) not the treatments.”*

### Weakness

Overall, 65% (13/20) of patients reported weakness, which they attributed to the treatment (10/13, 77%), the disease (5/13, 38%), and to surgery (3/13, 23%; 3/12, 25% of the patients who had also undergone surgery). Patients associated weakness with weight loss and reduced muscle strength. Some patients reported trying to restore their strength by increasing their physical activities. One patient reported the impact of weakness as follows: *“Yes. Yes*,* absolutely. I cannot (…) I can’t open things. I can’t put pressure (…) My arms are not nearly as strong*,* and that’s another reason why I’m trying to walk. I’m trying to build up my strength a little bit because I am so (…) I get so frustrated because I can’t do something. I can’t open something. I can’t tighten something. I can’t pick something up. It’s annoying.”*

### Diarrhea

In total, 75% of patients (15/20) reported diarrhea; most patients (11/15, 73%) attributed it to treatment, 13% (2/15) to surgery (2/12, 17% of patients who had also undergone surgery), and 7% (1/15) to the disease. Diarrhea also resulted in other signs/symptoms such as abdominal pain, dehydration, and fatigue. Diarrhea was described as one of the most bothersome signs/symptoms reported by some patients: *“I was on a chemo drug called irinotecan. The nickname for it is ‘I Run to the Can’ because it causes such severe diarrhea*,* and that really got me. And diarrhea*,* dehydration*,* fatigue.”*

### Vomiting

Vomiting was reported by 70% of patients (14/20) and attributed by patients to treatment (9/14, 64%), to the disease (2/14, 14%), and to surgery (2/14, 14%; 2/12, 17% of patients who had also undergone surgery). Severe nausea, increased sensitivity to odor, acid reflux, and early satiety were some of the factors that triggered vomiting in patients. Vomiting frequently occurred after treatment: *“I started vomiting out of the blue. I would just be sitting and then I’d vomit and then I’d feel fine (…) It was (…) emotionally*,* it was scary the first time*,* but I started experiencing more vomiting*,* I started to lose weight*,* I felt more weak. And I would say that lasted (…) When I first started chemo*,* I just started vomiting a lot*,* every day*,* sometimes twice a day. Every time I ate*,* I would vomit.”*

### Early satiety

Many patients (14/20, 70%) reported early satiety. Patients attributed early satiety to treatment (5/14, 36%), to surgery (5/14, 36%; 5/12, 42% of patients who had also undergone surgery), and to the disease (4/14, 29%). Early satiety was one of the signs/symptoms that led to a diagnosis of cancer in some patients: *“I started experiencing early satiety during meals as well as*,* ultimately*,* I started experiencing pain after meals. That led me to consult my primary care physician.”* Patients reported weight loss and changes in their eating habits, such as eating frequent, smaller meals, due to early satiety. Patients also linked early satiety to experiencing pain after meals.

### Difficulty in swallowing

Overall, 70% of patients (14/20) reported difficulty in swallowing; 43% of patients (6/14) attributed it to the disease, 43% (6/14) to treatment, and 29% (4/14) to surgery (4/12, 33% of the patients who had also undergone surgery). Difficulty in swallowing was one of the factors that led to a diagnosis of GC/GEJC for some patients and was one of the most disturbing signs/symptoms reported by patients: *“Yes*,* I started to (experience difficulty in swallowing) in the months leading up to the diagnosis (…) It’s not surprising because you had a golf ball-sized tumor right at the bottom of your esophagus.”*

### Taste alterations

Alterations in the sense of taste were reported by 70% (14/20) of patients and 79% (11/14) attributed these alterations to treatment. Taste alterations were rated by patients as one of the most disturbing signs/symptoms and they contributed to nausea, vomiting, and changes in diet. Patients reported having a metallic taste after chemotherapy or an altered sense of taste due to damage to their taste buds: *“The minute I got off of that drug*,* the oxaliplatin*,* it was fine. You’ll notice that anybody that complains (…) it tastes disgusting or nothing tastes good*,* that drug makes it happen.”*

### Abdominal pain

Many patients (13/20, 65%) reported constant stomach pain for a prolonged period before diagnosis; around half of patients (7/13, 54%) attributed it to the disease, 38% (5/13) to surgery (5/12, 42% of the patients who had also undergone surgery), and 8% (1/13) to the treatment. Patients referred to pain in the abdominal region as stomach pain, lower abdominal pain, and liver pain. Changes in bowel movements, such as diarrhea, constipation, and signs/symptoms like flatulence and bloating, caused abdominal pain in some patients. Stomach pain, often after a meal, was one of the more disturbing signs/symptoms reported by patients: *“Very painful. It was like a stabbing pain (…) constant pain that made it difficult to do anything.”*

### General pain

Overall, 60% (12/20) of patients reported general pain; 42% (5/12) of these patients attributed it to treatment, 33% (4/12) to disease, and 25% (3/12) to surgery (3/12, 25% of the patients who had also undergone surgery). Patients reported experiencing pain at the site of the tumor, pain due to radiation therapy, pain due to surgery, pain related to bloating and flatulence, pain in the anus due to diarrhea, and neuropathy-related pain. Patients did not report experiencing chronic pain; instead, they described the pain as usually acute, intense, and experience as ‘throbbing’ and ‘stabbing’: *“It’s like throbbing. I’ll get a throbbing (pain).”*

#### Impact-related concepts

Patients reported 31 impact-related concepts of aGC/GEJC during the interviews (Table 3). Most patients (90%) reported emotional disturbances linked to their experience with aGC/GEJC. Emotional disturbances, such as stress, worry, and anxiety, further contributed to patients’ inability to care for themselves, requiring patients to depend on assistance from others to engage in their daily activities (reported by 95% of patients). Being dependent on others, as well as no longer being able to care for their family members, had an emotional impact on patients. Moreover, 95% of patients mentioned that family members who cared for the patients experienced a significant burden.

**Table 3:** Impact-related concepts mentioned by patients during the interviews

Impacts	Total sample (*n* = 20)		Patient quotations
	Number of patients	Percentage of patients	
Role functioning			
Activities of daily living	18	90	*“So*,* when you are on a lengthy treatment break you gain energy*,* you gain*,* you know*,* your hair grows. You feel more normal. You have more energy*,* and you can perform activities like most people. However*,* when you are undergoing treatment*,* your life will be significantly impacted because the treatments are*,* I think*,* worse than the disease*,* and most patients will tell you that.”*
Recreational activities	17	85	*“On my activities. Yes. And just… Yeah*,* just not… The weakness and the tiredness impacting what I can do*,* and then the disease in general is impacting what I can do with my kids and just spur of the moment traveling stuff. I never know how I’m going to feel on a certain day. I’ll make plans*,* and then maybe I’m super tired that day or not feeling that great.”*
Work productivity	15	75	*“The diagnosis has been difficult with work just because I’m so exhausted. I’ve got extreme tiredness. I just like*,* after 4 or 5 hours*,* I just feel like I need to take a nap*,* so it has affected my work*,* and it’s affected my social life because I just don’t feel like going anyplace or doing anything. I’m just tired all the time. That’s been a really*,* really big impact.”*
Stopped working	11	55	*“Well*,* I had to quit my job at the beginning because I was vomiting all the time. It was just too unpredictable and I was weak; I just didn’t have energy. I’d have good days and bad days and it was too unpredictable. I loved my job. So*,* it affected it that way. I miss it.”*
Physical function			
Walking difficulties	12	60	*“Weakness*,* fatigue. Just the fact that you have a pump hooked up to you and that you have to learn how to sleep with it*,* you don’t feel as vital or energetic when you have*,* again*,* this pump connected to you for multiple days. And I went from playing basketball a month earlier to struggling to walk a lap*,* a mile lap*,* around the neighborhood and then having to take that really deliberately*,* really slowly.”*
Financial impacts			
Financial issues	13	65	*“Went back to work for a year*,* and then after that year they sat me down and they said*,* ’We’re going in a new direction*,* and we don’t know if you’ll be able to physically keep up with it’ (…) So they let me go. So*,* now I made a decision that I couldn’t go back to work. I ended up going out on Social Security disability*,* but the problem was I’m now making one-third of the money that I used to make*,* and I have the same bills (…) We had to declare bankruptcy.”*
Social impacts			
Requiring assistance	19	95	*“Well*,* you know*,* for example the house I live in we just did a little bit of remodeling*,* and so I will need help here and there on doing some of the physical work around here*,* or if there’s some yard work to be done I might need help on some of that. Anything real physical I definitely need help on still. I would also say a big thing that I need help on is childcare basically. If I’ve got both of my daughters*,* and I need to have someone help watch one while I work with the other*,* that sort of thing*,* I need help in that area too.”*
Family impacted	19	95	*“Horrible. Horrible. I am causing all of this traumatic experience to happen to my family. Horrible. Then my 21-year-old son was a heroin addict. Why do you think he was a heroin addict? Because of his mother telling him they’re only giving me 6 to 9 months to live*,* but I’m going to beat it. He’s watching all these physical things happen to me. He’s watching all these financial things happen to me.”*
Unable to take care of family members	10	50	*“Because I don’t have time to… If I’m tired and fatigued*,* I don’t spend time with my kids. They’re 4 and 8 now and they need me. They ask me*,* ’Mommy*,* can you come do this with us? Can we go play this? Can we go to the playground?’ I’m not capable of doing any of that.”*
Emotional impacts			
Emotional disturbance – non-specific	18	90	*“Of course. I don’t know if anyone could go through this and not be emotionally impacted by it. There were times early on*,* and in fact*,* every time there’s been a major setback… And there have been multiple major setbacks. At the beginning*,* when you’re in your mid-40s and all of a sudden… You think that you’re very healthy and strong*,* but you only have a year to live. I had panic attacks*,* like hyperventilating suddenly… And one of the things that I sort of vowed to myself that I was going to see my son graduate high school. He was in eighth grade when this started*,* and I’ve managed to do that. He’s a freshman in college now. But definitely a major sort of emotional impact in that… You do. You take stock of everything. You take stock of your life*,* what you’ve done*,* of your relationships*,* of how you are. The disease forces you to confront your mortality in a number of ways.”*
Worry	15	75	*“It has completely changed my life (…) When I was first diagnosed my immediate concern was for my daughter. I felt like*,* how could this be? How could I have cancer? I’m 37 years old and I have a daughter.”*
Stress	13	65	*“Right now*,* we’re in a little bit of a stressful stage because we’re waiting for my biopsy to come back. Waiting for test results. So that’s been our new life waiting for… We try to lead a pretty normal life until test results… we have to wait for those*,* so that (is a) waiting game.”*
Fear	12	60	*“Well*,* I mean*,* it’s just cancer as a whole*,* I mean*,* it’s huge. It’s emotional. It’s threatening in a lot of ways. Again*,* like I mentioned*,* to me I’m younger*,* and two young kids and a young family*,* and so it’s intimidating. I mean*,* cancer’s kind of a… I’m okay being a patient*,* but it is it’s intimidating and not knowing*,* I mean*,* no one can promise me any kind of outcome. I’m a little over*,* well*,* I’m a year and a half since the diagnosis of a cancer that was presented to me as pretty freaking daunting*,* so I didn’t even know if I would be here this year.”*
Change in appearance	12	60	*“It’s very bothersome to be honest. I’m dealing with it*,* but I don’t like it. I’ve always sort of enjoyed having a full head of hair. Most people do. I know for cancer patients it’s not even a gender thing*,* I mean*,* both men and women take a lot of… It’s about their identity.”*
Quality of life impacts			
Eating habits	17	85	*“Usually it’s after I eat something. It also depends on what I eat. Appetite and eating (have) been a challenge because of the fact that this is gastric*,* in my stomach. I’ve had to change to eating much more often and smaller meals and be very particular about what I eat. If I eat too much or if I eat something that I know is a trigger food*,* like gluten*,* dairy*,* those two in particular or raw vegetables*,* that’s another one*,* it will trigger this kind of… just this indigestion like you’ve eaten too much*,* and then it passes.”*
Changes in lifestyle	16	80	*“It has completely changed my life and significantly impacted the lives of those closest to me. I can no longer work. I can barely do housework and support my child. I can barely drive*,* so being independent has been very challenging.”*
Change in diet	14	70	*“Yeah*,* when I started the treatments back in July of 2019*,* I was on a lot of medications and all of them caused constipation*,* and so I had to do a lot of work. The laxatives weren’t doing anything. I was drinking coconut water. I was drinking prune juice. I was using suppositories (…) I have ways now to help. For example*,* when I’m on treatment I know I need to be drinking lots of water*,* lots of fruits*,* be eating lots of fibrous foods and that sort of thing. I guess*,* overall*,* I’ve learned that my body is just… Every body’s different*,* and for me I’m really sensitive to things that cause constipation.”*
Sleep disturbance	14	70	*“Well*,* I can tell you I think that it’s… I have it so often because when they did my surgery*,* the sphincter muscle that’s between your stomach and esophagus was obviously removed because that was the portion that was involved with cancer (…) When I lay down to sleep*,* it’s practically inevitable. I have to keep my bed propped up and sleep with my pillows. Again*,* it’s mostly a side effect of just not having that sphincter muscle (…) I know I’m supposed to lay on my right side as I sleep instead of my left*,* but often in my sleep*,* I’ll turn over and then I find myself in trouble. That’s probably my biggest long-lasting (side effect).”*
COVID-19-related impacts			
COVID-19-related impact	15	75	*“With my physician*,* I usually have… Right before every chemo*,* I have a medical visit. But because of COVID right now*,* it’s all been… I haven’t had an in-person visit in a long time*,* so it’s all via Zoom right now. She calls. We go over the last lab work*,* and then what happens is then the day before I go to chemo*,* I get my lab work. And if everything’s okay*,* then I get my chemo the next day.”*

The four most frequently mentioned impacts are described as follows.

### Emotional disturbances

After the diagnosis and during their experience of living with the disease, 90% (18/20) of patients reported being in a state of shock and experiencing other emotional disturbances such as frustration, confusion, dread, concern, or intimidation. Patients reported feelings of worry, stress, and fear caused by the burden of the disease on themselves and their family. Moreover, some patients described an increased sense of awareness of mortality that was imposed on them by having cancer: *“Of course*,* I don’t know if anyone could go through this and not be emotionally impacted by it. There were times early on and*,* in fact*,* every time there’s been a major setback… and there have been multiple major setbacks. At the beginning*,* when you’re in your mid-40s and all of a sudden… you think that you’re very healthy and strong*,* but you only have a year to live. I had panic attacks*,* like hyperventilating suddenly (…) You take stock of your life*,* what you’ve done*,* of your relationships*,* of how you are. The disease forces you to confront your mortality in a number of ways.”*

Patients also described emotional impacts related to side effects of treatment and to surgery, especially weakness, vomiting, and weight loss. Some patients experienced distress associated with the inability to perform simple actions, such as opening a can, because of physical weakness. Patients’ distress was also related to the changes in their appearance caused by weight loss and hair loss, and difficulty in accepting their new body image.

### Activities of daily living

Impacts on activities of daily living were mentioned by 90% (18/20) of patients and were mostly associated with signs/symptoms such as weakness, fatigue, and nausea. These were often related to treatment. With regard to impacts affecting recreational activities, patients reported having to cancel trips and vacations and no longer being able to play sports or to exercise owing to their low energy, fatigue, and nausea. The impact on recreational activities was attributed to both disease- and treatment-related signs/symptoms: *“So*,* when you are on a lengthy treatment break you gain energy*,* you gain*,* you know*,* your hair grows. You feel more normal. You have more energy*,* and you can perform activities like most people. However*,* when you are undergoing treatment*,* your life will be significantly impacted because the treatments are*,* I think*,* worse than the disease*,* and most patients will tell you that.”*

### Requirement for assistance

Most patients (19/20, 95%) reported requiring assistance in their daily lives. This was related to patients experiencing difficulty conducting physical work and their need for help with activities of daily living and taking care of family members. Patients also reported the need to have an acquaintance drive them to the infusion center to receive treatment and to involve their family in the provision of care: “*Well*,* you know*,* for example*,* the house I live in*,* we just did a little bit of remodeling and so I will need help here and there on doing some of the physical work around here*,* or if there’s some yard work to be done*,* I might need help on some of that. Anything real physical I definitely need help on still. I would also say a big thing that I need help on is childcare (…) If I’ve got both of my daughters*,* and I need to have someone help watch one while I work with the other*,* that sort of thing*,* I need help in that area too.”*

### Impact on family

Most patients (19/20, 95%) reported that their family members were impacted by their disease and suffered from emotional disturbances following the patient’s diagnosis. According to patients, family members would often become their caregivers and, in some cases, they would stop working to become full-time caregivers. One patient described the situation as: *“Horrible. Horrible. I am causing all of this traumatic experience to happen to my family. Horrible. Then my 21-year-old son was a heroin addict. Why do you think he was a heroin addict? Because of his mother telling him they’re only giving me 6 to 9 months to live*,* but I’m going to beat it. He’s watching all these physical things happen to me. He’s watching all these financial things happen to me.”*

### Other impacts

Cognitive impacts affected a minority of patients and included dizziness, confusion, cognitive problems, speech difficulties, and the inability to read. Patients also reported impacts on their lifestyle, their work, their financial situation, and their ability to care for others: *“It’s affected a lot. My kids… I have two kids and since I’m not capable of taking care of them*,* my parents are taking care of them. So*,* they’re overwhelmed as well. My Dad had to quit working so he could take the kids to school (and) from school. Of course*,* thank God*,* my Mom is still on unemployment*,* so she’s helping as well for the moment. But it’s been tough. I have family members that (cook) for me*,* someone comes and brings me something*,* makes sure that I’m not alone. It’s just (that) life is not the same anymore.”*

#### Refinement of the conceptual model

**Fig. 3:**
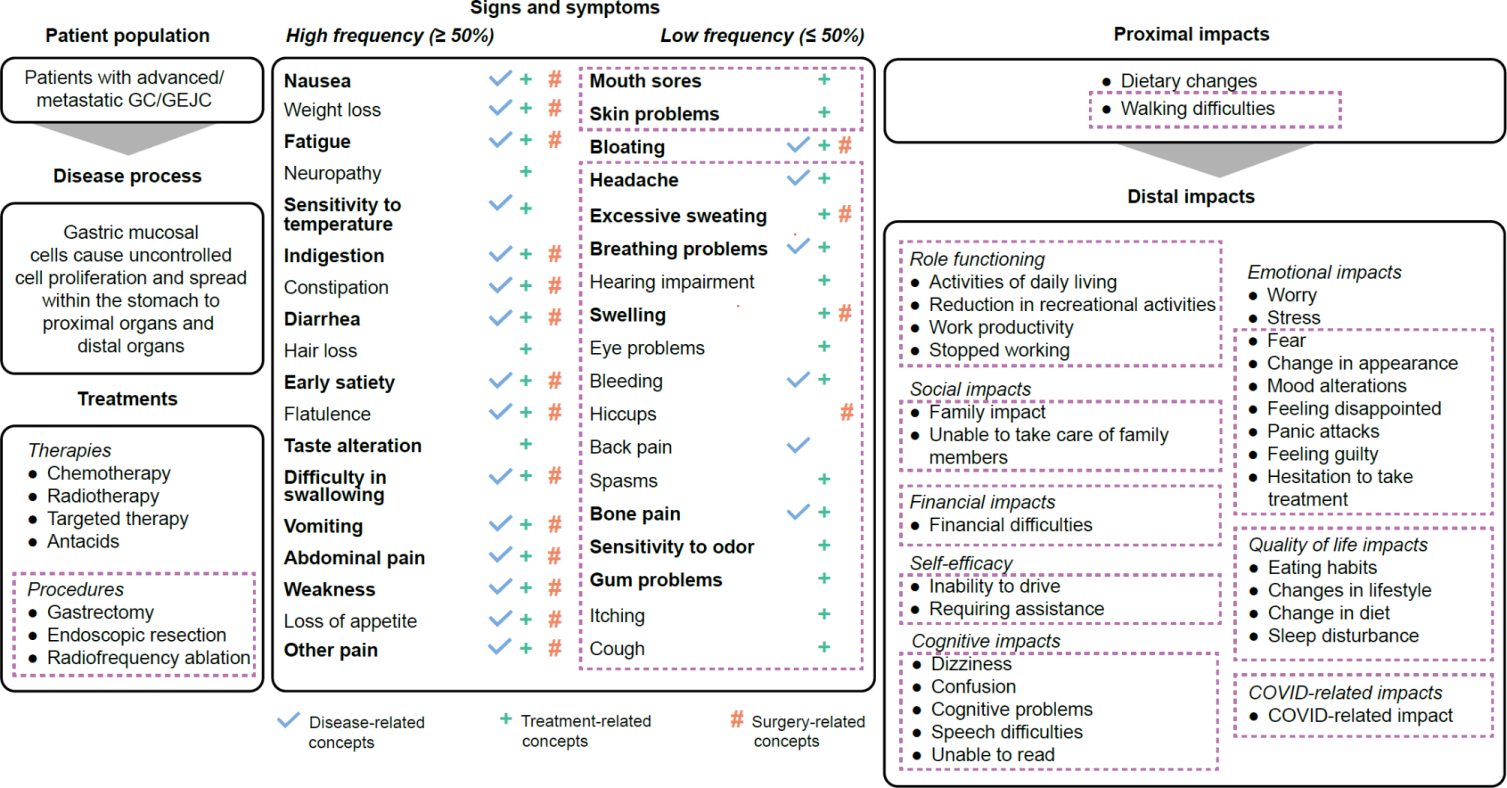
Final conceptual model Bold text in ‘Signs and symptoms’ indicates high disturbance (disturbance rating > 5 on a scale of 0 to 10, with 0 = not disturbing at all and 10 = extremely disturbing. Changes from the preliminary conceptual model to the final conceptual model are indicated with boxes with dashed lines. In the final model, signs/symptoms were split into surgery related and non-surgery related. Impacts that are directly related to the disease are defined as proximal impacts, while distal impacts are secondary impacts COVID, Coronavirus Disease; GC, gastric cancer; GEJC, gastroesophageal junction cancer

The preliminary conceptual model that was developed based on data from the six studies was refined based on insights from patient interviews, as described in Fig. 3. The updated conceptual model included whether signs/symptoms were attributed to the disease, treatment, and/or surgery. All concepts identified in the patient interviews were added, regardless of the number of mentions. Signs/symptoms that occurred in ≥ 50% of patients in the TLR were consistent with those identified in the interviews, with the exception of bloating, which was moved from high to low frequency in the updated conceptual model. The updated conceptual model also included all low frequency symptoms identified during the interviews and their attributions to either the treatment, the disease and/or surgery. Like the preliminary model, the updated model distinguished between proximal and distal impacts; however, in the updated model, these were restructured and additional domains and impacts were added (Fig. 3 and Supplementary Fig. 1).

### Treatment expectation for new trials

To understand patients’ expectations and preferences for any new treatments or for clinical trials, interview questions focused on patients’ willingness to enroll in clinical trials, their perception of study procedures, and their views on the development of new treatments.

#### Willingness to enroll in a clinical trial

The majority of patients (17/20, 85%) reported that they would be willing to enroll in a clinical trial, two patients (2/20, 10%) were unsure, and one patient (1/20, 5%) stated that they would not enroll. For some patients, the willingness to participate in a clinical trial depended on whether or not they perceived that as a way to potentially extend their life expectancy: “*It’s all a matter of risk versus benefit. Definitely for me*,* if the benefit was improving my longevity significantly*,* definitely I’ll be interested.*”

Patients’ willingness to enroll in a trial was also dependent on receiving information about the best available treatment: *“I would have to be very well informed before going off any treatment in order to exclusively go on a trial.”*

Six patients (6/20, 30%) reported that they had previously participated in a clinical trial. When commenting on their experiences, some patients reported that they had needed to leave the trial after their scans showed that the treatment was not working, while other patients reported that the trial had stopped the progression of their cancer. One patient (1/20, 5%) who had an unsatisfactory outcome from a clinical trial reported that this did not significantly change their willingness to enroll in a future trial: *“Even though the medication itself didn’t work out for me*,* I have always been eager and interested in participating in clinical trials because I’m just always attracted to the idea of cutting-edge medication.”*

Patients expressed the wish to have better access to clinical trials, commenting that they would prefer: *“Having them accessible in various locations.”*

The patient who said they would not participate in a clinical trial stated that this was owing to the possibility of being enrolled in the placebo group: *“If I had a chance that I was going to get a placebo*,* I wouldn’t enter it.”*

#### Perception of study procedures

Patients believed that if questionnaires and interviews were helpful to the doctors and scientists, and to other patients experiencing the same disease, they should complete them. This is illustrated by this patient’s comment: *“I think the more data that the medical field has*,* the drug companies*,* the doctors*,* I think it’s more important on the whole so they know what’s going on…But I do think*,* as a patient*,* you can’t withhold anything. It should maybe be explained like: ‘You would have to do this and that and answer.’ You should answer your questionnaires and you should always be truthful (about) what’s going on.”*

Most patients expressed a willingness to accept a new tumor tissue biopsy, both to assess a specific biomarker and for enrollment in a trial where they would receive a treatment with fewer side effects. However, two patients (2/20, 10%) stated that they would refuse to get a biopsy for the purpose of a biomarker assessment or trial owing to the repetition of biopsies in the past that they considered to be unnecessary, as well as concerns about the safety of the procedure. One patient stated their preference regarding which type of biopsy they would be willing to have: *“I might prefer a liquid biopsy*,* being that it’s just taking blood.”*

#### Development of new treatments

Three patients (3/20, 15%) expressed a desire for new treatments to be developed with fewer side effects (e.g., nausea, neuropathy) compared with traditional chemotherapy. One patient commented: *“Well*,* the most important thing for my treatment… Well*,* there would be two most important things. First of all*,* that it’s effective for me and*,* secondly*,* that the side effects are minimal.”*

Some patients reported that they would prefer to be included in a trial focusing on an innovative therapy rather than on a chemotherapy trial: *“I’m more interested in new therapies*,* like gene therapies or CAR-T therapies or – I don’t know – anti-angiogenesis*,* all the cutting-edge stuff now because a lot of these systemic things that have been tried*,* I’ve done them.”* Two patients (2/20, 10%) stated a preference for a pill formulation compared with an infusion.

### Experience of care

Patients were asked to describe their experience of care, which included their experience with medical visits, disease monitoring, systemic therapy, and surgery, as well as their interaction with medical professionals. Most patients reported a positive experience of care, whereas some patients reported negative experiences, such a late diagnosis or misdiagnosis (for more information see Supplementary information, Patients’ experience of care from health care providers).

#### Impact of medical visits

Overall, the frequency of medical visits was not a major concern for most patients. However, frequent medical visits were challenging for patients who required assistance to travel and/or needed to travel long distances: *“…that was a pain to find an oncologist available within a reasonable distance. The first one we found was an hour away from home*,* so we started with that*,* and then we got transferred to another one that was about 45 minutes away.”*

#### Impact of disease monitoring

Monitoring of disease conditions through laboratory tests, computed tomography (CT), and magnetic resonance imaging scans was generally accepted by patients. One patient expressed their willingness to undergo frequent monitoring in order to understand how effective the treatment was: *“I would want more monitoring because I would want to see*,* if I’m on a treatment*,* if the treatment’s working*,* (or) if it’s not working.”*

However, some patients were not willing to undergo frequent scans and laboratory tests owing to the associated costs, the discomfort related to traveling to test centers, and the need for assistance to reach the test centers. This quotation demonstrated one patient’s awareness of both the benefits and the high cost of frequent testing: *“What will really tell whether it’s working or not is the CT scan and*,* again*,* we are going to do that next month. I think that’s all good to monitor what’s happening from a few different angles. (…) I have to consider CT scans*,* and blood labs*,* and all these other things that come along…*,* and those are all costly.”*

Having to undergo a biopsy was considered acceptable for some patients and bothersome for others. Most patients were aware that a biopsy was the main procedure to confirm the diagnosis and determine the type of mutation that was present, which would then enable the patient to receive targeted therapy. One patient stated: *“They did biopsy that and that’s what led to the diagnosis as soon as I was biopsied.”*

#### Impact of systemic therapy

Patients received multiple types of systemic therapy: most patients received chemotherapy (*n* = 17, 85%), while half of the patients received therapy (*n* = 10, 50%). Chemotherapy was often administered as intravenous infusions, although some patients found having the infusion administered through ports more convenient. Patients also described how receiving treatment affected their usual lifestyle both by becoming a priority in their schedule and introducing challenges to their routine: *“Well*,* the priority for me has been anything related to my infusion schedule or doctors’ appointments. Those take priority*,* and at this point I know that when I go in for infusion*,* I don’t schedule anything for myself for a whole week.”*

All patients reported that chemotherapy caused multiple side effects, especially nausea. *“I had significant nausea and vomiting… even from the very first cycle. I felt bad anyway*,* and then the chemo made me feel worse. (…) I have significant reflux disease. When I have an episode of that*,* it results in nausea and often vomiting.”*

To counteract the chemotherapy-related side effects, patients received other medications such as antinausea pills, steroids, vitamin B12 shots, pain medications, laxatives, and iron supplements. However, these medications often had side effects themselves, such as constipation deriving from antinausea pills. Due to intolerable side effects or poor efficacy of chemotherapy, some patients received immunotherapy after chemotherapy, while other patients received immunotherapy along with chemotherapy. Patients reported that immunotherapies had fewer side effects and were easier to receive than conventional chemotherapy.

#### Impact of surgery

Twelve patients (12/20, 60%) underwent surgery. Patients reported very severe complications related to surgery. Some patients attributed side effects, such as early satiety, difficulty in swallowing, dehydration, indigestion, weight loss, and vitamin B12 deficiency, to a partial or total gastrectomy. Two patients reported facing severe issues related to eating and drinking after the surgery: *“After the surgery*,* I don’t have a sphincter between my stomach and esophagus*,* so it’s not just reflux. Sometimes I would just regurgitate*” and *“I never have an appetite*,* since I had most of my stomach removed.”*

Some patients had multiple complications following surgery, such as infection: *“The stomach was gone*,* but the surgery was brutal. It was a 13-hour surgery. (…) a 13-hour surgery is a long time so your body is starting to shut down some of the organs. One of the lungs for breathing collapsed and didn’t come back*,* so several complications. The junction where the tube was done was leaking*,* so I had to go back in surgery shortly after that to put in a stent*,* and we did that a few times. So basically*,* it was 3 months without eating or drinking anything. There was also infection internally*,* and the reactions to the antibiotic that was used at that time causing even more infection.”*

## Discussion

This study combined the results from a TLR and the concept elicitation interviews with data from previous qualitative research to identify signs/symptoms and impacts associated with aGC/GEJC and its treatment, as well as the impact of surgery on the patients interviewed who had undergone surgery, which was the majority. The diversity and number of concepts collected suggested that there was heterogeneity in the experiences reported by patients. Despite this, saturation of signs/symptoms and impacts was achieved in the fourth and final wave of interviews. This information was used to develop and refine a conceptual model for aGC/GEJC. Taken together, the results from the patient interviews show how one or more PROs can provide an excellent and comprehensive understanding of the patient experience, highlighting how the new conceptual model can provide a basis from which to appraise existing PROs for their conceptual comprehensiveness.

The most common signs and symptoms of aGC/GEJC were nausea, fatigue, and weight loss, which were reported by all patients. Of these, nausea and fatigue were both rated as being disturbing. Other frequent and disturbing symptoms included temperature sensitivity, indigestion, diarrhea, weakness, early satiety, taste alterations, difficulty in swallowing, abdominal pain, vomiting, and other pain. Signs/symptoms that had a high disturbance rating but were not frequent enough to be considered most important included breathing problems, bone pain, and sensitivity to odor. Overall, the high frequency signs/symptoms identified in the TLR were all identified as being frequent in the patient interviews (defined as occurring in ≥ 50% of patients in both the TLR and the interviews), with the exception of bloating, which was not categorized as frequent in the interviews. No additional symptoms were captured by the interviews that had not already been identified by the TLR.

Patients’ attribution of many signs/symptoms to surgery emerged in the interviews and not in the TLR. While the majority of signs/symptoms were attributed to treatment, some signs/symptoms, such as hiccups, indigestion, abdominal pain, early satiety, and loss of appetite, were attributed to surgery by around one-third to one-half of patients. These results help provide a better understanding of the burden of surgery and treatment compared with the burden of disease. To reduce the burden of surgery-related signs/symptoms, less invasive surgery, which can preserve some stomach function, may be preferable to more radical approaches, meaning that early disease detection is imperative.

Requiring assistance, impacts on the patient’s family, emotional disturbances, and impacts on activities of daily living were the most common impacts reported by patients during the interviews. Treatment was a major burden to patients and was linked to the side effects of chemotherapy and the inconvenience of the treatment schedule. Surgery was also a major burden, related to complications such as disrupted sleep or not being able to eat normally. These most common impacts were also identified during the TLR and are captured in the final conceptual model. The patient interviews also identified additional impacts, such as the inability to drive, and provided further details, which were captured in the final conceptual model. For example, depression was expanded into a more detailed list of emotional impacts based on the patient interviews, such as fear, change in appearance, mood alterations, feeling disappointed, panic attacks, feeling guilty, and hesitation to take treatment.

Most patients provided positive feedback on their experience of care, highlighting the importance of the relationship between health care professionals and patients who are receiving cancer care. However, some patients reported receiving a misdiagnosis or a late diagnosis, which has a major impact on prognosis in GC/GEJC, especially if the cancer could have been diagnosed before it has advanced or metastasized [18]. PED are important to understand the early signs of the disease; knowledge of these early signs increases the likelihood of a prompt diagnosis, which in turn improves patients’ survival outcomes and HRQoL. Finally, patients reported having to travel long distances to attend medical visits, which highlights the need to facilitate patients’ access to specialized hospitals to support the early diagnosis of aGC/GEJC.

When considering clinical trials, 85% of patients expressed a willingness to enroll in a trial, motivated by the prospect of receiving innovative and effective treatments with fewer side effects than chemotherapy. However, only 30% of patients had previously participated in a clinical trial. Poor access to clinical trials was a major reason for the lack of participation in clinical trials, alongside the possibility of being included in the placebo group. Indeed, access to clinical trials varies substantially in both rural and urban areas in the USA [19, 20]. These results highlight the need to improve access to oncology trials, which is crucial to achieve health equity [21]. ‘Decentralization’ of clinical trials using digital medical devices and electronic medical records/platforms could help to make them more accessible [22]. The insights on clinical trials captured from patients in this study could be used to improve patients’ access to trials and clinical trial protocols.

PED from this study provide valuable, in-depth information that can help improve our understanding of the impact of aGC/GEJC and support clinical trial design and PFDD. These data can also help characterize the disease, select clinical trial endpoints and determine how these endpoints can be used to measure the benefit of treatment in the context of clinical studies. The FDA has provided guidance on how to identify what is important to patients, and to incorporate PED into clinical trials [23]. In addition, involving patient advocate groups across the clinical trial life cycle can help support PFDD. For example, the National Cancer Institute’s National Clinical Trials Network has a long history of including patient advocate feedback in the development of concepts to be measured in clinical trials [24].

A limitation of the study was that the TLR was completed in 2021; however, this was because the TLR was used to inform the conduct of the interviews, which were conducted following the TLR and provided updated information on aGC/GEJC. There is limited research in this area so it is unlikely that there are more recent studies that would add to these findings. Another limitation was that the qualitative research sample size was small (*N* = 20), although this is not uncommon for concept elicitation research [25, 26] and the sample size was in line with recommendations for sample sizes needed to achieve saturation [16]. Indeed, concept saturation was reached during the interviews, confirming that the sample size was sufficient to capture the experience of patients with aGC/GEJC [16, 17]. In addition, the methods used to assess the literature aligned with those used by similar studies [17]. Finally, there was a lack of cultural diversity, with all patients recruited from the US and 19 out of 20 patients being White. Lack of diversity in clinical studies has long been recognized, with the study populations for cancer trials often not matching the composition of the target population [21]. To address the lack of diversity in clinical research, the FDA has issued draft guidance for a Diversity Plan that provides a framework focused specifically on racial and ethnic characteristics [26]. However, the qualitative sample was diverse in terms of disease characteristics and treatment history, and further research into these differences and their impact would provide a better understanding of the patient experience and unmet need.

## Conclusions

This primary and secondary qualitative research study generated a comprehensive description of the most prevalent and disturbing signs/symptoms and impacts of aGC/GEJC. The resulting conceptual disease model can guide the clinical outcome assessment strategy for the development of innovative treatments, in line with PFDD and patient-focused medicine development initiatives.

## Electronic supplementary material

Below is the link to the electronic supplementary material.

Supplementary Material 1

Supplementary Material 2

## Data Availability

The datasets used and/or analysed during the current study are available on reasonable request. Details on Sanofi’s data sharing criteria and process for requesting access can be found at https://vivli.org/.

## Abbreviations

COVID: Coronavirus Disease
COVID-19: Coronavirus Disease 2019
CT: computed tomography
GC: Gastric cancer
GEJC: Gastroesophageal junction cancer
EORTC: European Organisation for the Research and Treatment of Cancer
FDA: US Food and Drug Administration
IRB: Institutional Review Board
NA: Not applicable
NP: Not provided
HRQoL: Health-related quality of life
PED: Patient experience data
PFDD: Patient-focused drug development
QLQ-C30: Quality of life questionnaire-C30
QLQ-STO22: Quality of life questionnaire-STO22
SD: Standard deviation
TLR: Targeted literature review
WCG-IRB: WIRB-Copernicus Group
WIRB: Western Institutional Review Board
