# Supplementary material for: A conceptual model for advanced/metastatic gastric or gastroesophageal junction cancer: a review of qualitative studies and results from patient interviews
Source: BMC Cancer. 2025 Jan 15;25:88. doi: 10.1186/s12885-025-13474-9 (PMC11734369; doi:10.1186/s12885-025-13474-9)
Supplement: Supplementary file 2 — Supplementary Material 2 [file 12885_2025_13474_MOESM2_ESM.docx]

# **A conceptual model for advanced/metastatic gastric or gastroesophageal junction** cancer: a review of qualitative studies and results from patient interviews

# Supplementary information

## Additional file 1

### Interview guide summary

The four main topics indicated by the guide were:

1. Disease background and treatment history:
   1. experience at diagnosis, prior and during therapy
   2. experience with or without surgery
   3. perceived effectiveness of the treatments and their side effects.
2. Current disease and treatment experience:
   1. experience and disturbance ratings of signs/symptoms of advanced/metastatic gastric cancer/gastroesophageal junction cancer (aGC/GEJC)
   2. experience of impacts of aGC/GEJC
   3. experience of patients’ family and caregivers.
3. Experience of care:
   1. frequency of medical visits and tumor assessment
   2. mode of therapy administration
   3. treatment regimen schedule
   4. interference with daily/weekly activities
   5. quality of interactions with health care professionals
   6. family/caregivers’ experience with care.
4. Treatment expectations:
   1. characteristics of new treatments
   2. patients’ willingness to enroll in clinical trials (including trials with reversible visual side effects)
   3. duration of enrollment and treatment regimens
   4. patients’ willingness to complete questionnaires at regular intervals and to participate in entry/exit interviews.

### Patients experience of care from health care providers

Patients provided six positive, two negative, and two neutral quotations about their experience with care from health care providers. Positive feedback was mostly related to the efficiency, knowledge, punctuality, and positive attitude of hospital staff. Patients also appreciated the comfort and privacy during treatments and the quality of the hospital facilities. One patient reported: *“Excellent. Top notch, excellent. They are… I mean, not just their skills, but they’re just the most caring people.”*

Negative feedback was mainly related to late diagnosis, inefficiency of staff in infusion administration, unavailability of familiar medical staff, physician’s negative attitude towards treatment, and unfriendly nature of medical professionals: *“It wasn’t handled great. I think it was just my gastroenterologist. The only one I’d ever dealt with. Finally went to him for the upper scope. He didn’t have the best bedside manner. He just said ‘We found something on the scan and it’s not good. It’s cancer.’ It was just real straight forward. It could have been handled better, I think.”*

These results show that most patients’ experience of care was positive overall. However, patients complained about receiving late diagnoses, frequent changes in hospital staff, and the poor bedside manners of some health care professionals.

## Additional file 2

### Supplementary tables

Supplementary Table 1 Eligibility criteria

| **Inclusion criteria** | **Exclusion criteria** |
| --- | --- |
| - Age 18 years old or older - Able to communicate proficiently in English - Confirmed diagnosis of aGC/GEJC by a physician based on histological/radiological investigations - Willing and able to participate in the study - Legally and mentally capable of providing informed consent to research - Willing and able to participate in a 75- to 90-minute interview to discuss signs, symptoms and impacts related to their experience with GC and GEJC - Resident in any state in the USA (Puerto Rico was excluded) | - A mental disability or significant mental illness, legal incapacity or limited legal capacity, or any other lack of fitness, which, in the opinion of the screener, would preclude participation in or ability to complete the study - Unwilling to allow physician verification of health records |

aGC/GEJC, advanced/metastatic gastric cancer/gastroesophageal junction cancer.

**Supplementary Table 2** Overview of studies used to develop the preliminary conceptual model

| **Study** | **Study type** | **Study objective** | **Population** | **Geographic location** |
| --- | --- | --- | --- | --- |
| Lin et al., 2020  Journal article | Qualitative study | To describe multiple co-occurring signs/symptoms, symptom experiences, and symptom self-management strategies in patients with GC | Patients (n = 10) with advanced GC (Stage III–IV). All participants received chemotherapy, whereas 3 patients had undergone surgery also | USA |
| Martin et al., 2017  Conference presentation | Qualitative study interviews were conducted in 2 parts  *Part A*: open-ended interview to understand general patient experience  *Part B*: module-based interview using EORTC questionnaire probes QLQ-STO22 followed by QLQ-C30 | To understand the key disease- and treatment-related signs/symptoms that are reported by patients with GC/GEJC and determine key PRO concepts that should be measured in clinical trials | Patients (n = 12) with unresectable, locally advanced or metastatic GC/GEJC | USA |
| Morlock et al., 2017  Conference presentation | Literature review of patient blogs/oncologist insights and conceptual model development | To identify signs, symptoms and impacts of advanced esophageal cancer, GC and GEJC and map these to the PRO instruments frequently used to assess patient experience in clinical trials and the real world | Patients with GC, GEJC, and esophageal cancer and oncologists’ validation of concepts | USA |
| Morlock et al., 2018 Conference presentation | Qualitative study | To assess and measure the signs/symptoms and impacts of locally advanced nonresectable/metastatic GC or GEJC reported by recently diagnosed patients in the USA | Patients with advanced/metastatic inoperable GC (n = 5) and GEJC (n = 6) | USA |
| Humphrys et al., 2020 [1][1]Conference presentation | Patient survey, secondary care case note review, and qualitative study (semi-structured patient interviews) | To explore patient’s understanding, experience, and presentation of signs/symptoms before diagnosis | N = 127 patients with esophageal cancer (n = 102) and GC (n = 25). Of these, 26 patients participated in an additional face-to-face interview | UK |
| Uribe et al., 2019 [5]  Journal article | Qualitative study | To describe the access barriers to palliative care perceived by adults with GC, caregivers and physician in Santander, Colombia | N = 56; n = 14 adults diagnosed with GC, n = 24 caregivers, and n = 18 physicians | Colombia |

EORTC, European Organisation for the Research and Treatment of Cancer; GC, gastric cancer; GCJE, gastroesophageal junction cancer; PRO, patient-reported outcome; QLQ-STO22, quality of life questionnaire-STO22; QLQ-C30, quality of life questionnaire-C30.

Supplementary Table 3 Disease and treatment-related signs/symptoms of aGC/GEJC derived from the TLR

| **Disease-related signs/symptoms** | **Both disease- and treatment-related signs/symptoms** | **Treatment-related signs/symptoms** |
| --- | --- | --- |
| - Dysphagia - Abdominal pain - Pain in tumor sites - Neuropathy - Bloating/abdominal pressure - Regurgitation of acid/bile/food - Flatulence - Early satiety - Discomfort when eating - Pain, general - Stomach discomfort - Bleeding in stomach - Belching - Heartburn/indigestion - Gastritis (burning sensation) | - Fatigue/tiredness - Weakness - Gastrointestinal signs/symptoms   - Nausea   - Diarrhea   - Vomiting   - Constipation - Flu-like signs/symptoms - Abdominal swelling - Loss of control of bowel movements/urination - Voice changes - Coughing - Bloody stools | - Lack of appetite - Weight loss - Sensitivity to temperature - Taste alterations - Neuropathy - Mouth sores/pain in mouth - Pain in the stomach that moves to back and legs - Chest pain - Back pain - Gum pain/problems - Changes in skin (red spots/peeling) - Sensitivity to spicy food - Cramping - Swelling in legs - Shortness of breath - Dizziness - Dry mouth - Hair loss - Toenails splitting - Nails separated from nail bed |

aGC/GEJC, advanced/metastatic gastric cancer/gastroesophageal junction cancer; TLR, targeted literature review.

Supplementary Table 4 Impacts of aGC/GEJC derived from the TLR

| **Proximal impacts** | **Distal impacts** |
| --- | --- |
| - Dietary changes - Impact on activities of daily living - Sleep disturbance - Excessive sleeping - Trouble enjoying meals - Taking a long time to complete meals - Trouble eating in front of others - Difficulty taking in solids/liquids - Psychological pressure while eating - Difficulty concentrating - Difficulty remembering things - Impact on physical functioning (inability to walk, trouble doing strenuous activities) | - Social impacts   - Impact on social functioning   - Inability to participate in usual activities   - Need for additional support in managing signs/symptoms   - Impact on family/relationships   - Lack of desire to do social things   - Impact on sexual life - Self-efficacy   - Need for support from clinical team   - Challenges with medication management   - Lifestyle modifications   - Need for additional support in managing signs/symptoms   - Need for psychosocial and spiritual support - Work impact   - Impact on work   - Financial impact - Emotional impacts   - Depression   - Anxiety   - Worry   - Feeling tense   - Moodiness   - Stress   - Feeling isolated   - Feeling confined to home   - Feeling irritated   - Desire to look for positive meaning in patient’s life |

aGC/GEJC, advanced/metastatic gastric cancer/gastroesophageal junction cancer; TLR, targeted literature review.

Supplementary Table 5 Quotations about signs/symptoms of aGC/GEJC from the patient interviews

| Symptom | Quotations from patients |
| --- | --- |
| Nausea | - *“I’ve only had one bout of vomiting, which actually just happened last week. And then the nausea, yes. I would say it’s all related to food, so I have to be real careful not to eat too much at a time because I get to a point where then I will probably throw up.”* - *“I had significant nausea and vomiting and even from the very first cycle. I felt bad anyway, and then the chemo made me feel worse. (…) I have significant reflux disease. When I have an episode of that, it results in nausea and often vomiting.”* - *“And the nausea was the worst. It was about… there was 2 days where it was the worst, and that was the day they take (…) that off and the next day were the worst, and then it got better over the next 2 days and pretty much dissipated. (…) The sense of smell, this heightened sense of smell, especially towards food, coupled with the nausea created this huge lack of appetite.”* - *“The nausea was real bad the day of the treatment in the evening. The first time and the second time it was real bad the day after, and then it cleared up, so it didn’t last long either time.”* - *“Really, it’s just the infusion, and if I happen to have some heavy nausea. (…) It can last all day pretty much, and when you’re on the treatment.”* - *“Right after my first treatment I started exhibiting signs/symptoms… nausea, vomiting.”* - *“I did [experience nausea] with irinotecan.”* - *“The antinausea medication they give me at infusion, one of them will last 5 days, and another one will last 8 hours. Again, the reason I’m so hesitant on taking those because one of the side effects of the antinausea is constipation.”* - *“I have a lot more nausea from the treatment. And also I think I mentioned I have this super heightened sense of smell, so food smells make it very unappealing.”* - *“I would say it’s a 10 too because I just can’t stand being nauseous because it affects my eating.”* |
| Fatigue | - *“But the fatigue is just that, kind of a mental lack of, or reduced energy and motivation... I want to say general fatigue, but a little lower energy, and I wouldn’t doubt that some of that, if not most of it, is due to my not full eating potential.”* - *“Fatigue? I’ll just be… it’s a strange… It’s not like I’m tired and I’m going to go take a nap. I’ll be sitting maybe on the couch and I’ll just all of a sudden realize… I’ll wake up and realize I had fallen asleep. It’s a weird… it’s strange. It’s not … normal. Before cancer, gee I’m tired, I need a nap for a little while and then I wake up refreshed.”* - *“It started with I was very fatigued, and I was still working … I was extremely anemic… I was very tired. I had a lot of nausea.”* - Moderator: “We talked about the fatigue. Can you tell me on a day that it’s at its worse, what is it like when you’re tired?   - *“At its worse, I just don’t get out of bed for 30 plus hours. I just sleep most of the time and that’s about it at its worst.”* - *“Every day I definitely feel some fatigue, but it’s definitely, I would say, worse for about 3 to 4 days after my chemotherapy. (…) I have no muscle mass right now, so that… I kind of… I think I’d lump that in with fatigue, but yeah, I’m definitely weak compared to what I used to be.”* - *“Fatigue, yeah, but the fatigue is related primarily to the impact of the treatments and not the disease itself.”* - *“I think it’s more the disease because I had the fatigue before I started the chemo. It just seems to be more consistent now that I had started the chemo. The fatigue came on in and out of the operating room, in and out of the hospital, it’s like I never could build my strength back up after the first round of surgery and just them messing with my stomach area, and then the chemotherapy just made it even more pronounced and worse. It’s like I have no energy for nothing.”* - *“I guess I would say the fatigue. Fatigue is the number one (…) I couldn't eat a thing. I couldn't drink for 6 weeks, I couldn't do any (thing).”* - *“I would say it’s a 5 at all times. I am constantly tired, constantly tired.”* |
| Sensitivity to temperature | - *“[Sensitivity] to hot and cold. No. That was just with the oxaliplatin that I was getting at the time that was a cold sensitivity.”* - *“I have had that with my first treatment. I did have sensitivity to cold. But after I discontinued that treatment, I haven’t had that.”* - *“That would begin right after treatment. And it was mainly I couldn’t drink cold liquids. And I couldn’t… if I went to get something out of the freezer, it was like feeling frostbite. So I’d put gloves on to get things out of the freezer. That lasted a couple of days after the treatment, it wasn’t during the whole treatment. I’d go get another treatment and it would happen again for a couple of days.”* - *“I’m not supposed to drink anything extremely cold. I’m not supposed to drink anything extremely hot. I do have my coffee in the morning, and it doesn’t bother me. Cold bothers me more than hot except in the summer. I can drink a cold beer. I can’t guzzle… never guzzle again. I sip. I can never ‘glunk-glunk’ again. I don’t know, without a stomach you just can’t do that, so I sip.”* - *“The cold sensitivity when it was happening was the worst, I think.”* - *“Most bothersome for me is the neuropathy cold sensitivity. That one is the constant. It never goes away.”* - *“Very bothersome… 10. It was painful. Painful to drink a cold glass of water.”)* |
| Indigestion | - *“Actually, the one pill that I take is a little, teeny famotidine pill. It’s so little, it’s like a pin prick. (…) I also did lose to the surgery the portion of your stomach that does that, the digestive juices and things like that.”* - *“I have significant reflux disease. When I have an episode of that, it results in nausea and often vomiting. Again, I know what it is. It's reflux. It comes and goes, probably on an average of four nights a month.”* - *“I have a lot of acid reflux. Well, it’s not acid reflux. I have to take that back. I have bile reflux because I don’t have a stomach, so what the gastroenterologist told me is happening is that the bile backs up into my esophagus, so I have a medication that pushes that back down and helps tremendously to take care of that… (It’s related) to the surgery (…) not the treatments.”* - *“I cannot sleep. Because I don't have the stomach, I can't sleep entirely prone. I have to be elevated a little bit, because that little muscle that you have isn't there, so stuff will come up. It's not like acid reflux, it just comes up because there's nothing to stop it. I do not sleep in a prone (position), I have to have at least a couple (of pillows).”* - *“Yes, it wakes me up. I’m kind of choking on it, so I end up vomiting and coughing for an extended period of time.”* - *“It happens every once in a while. Not all the time anymore because I’ve changed my diet completely since diagnosis. I’m eating a lot better. I’m eating specific foods to help with the pH balance of the stomach, and all that sort of thing. I do drink coffee still, and I know that’s very acidic, so I have to monitor that. So on particular days if I have a little too much I can feel it.”* - *“One of the reasons I did decide to do an endoscopy, when the cancer was discovered, was because I’d been having acid reflux occasionally. It had gone from instead of like once or twice a month to once or twice a week.” “It’s like a constant irritation in your throat around where you’re… deeper in your throat where you think of your Adam’s apple being on the trachea. And it’s like a lot of times when you swallow, it’s accompanied by a wince.”* |
| Weakness | - *“I would say both but probably… I would say both. Yeah. Because I know the treatment’s weakening me, but I know the disease is weakening me by not being able to eat. So I would say both. Yeah.”* - *“I think it’s the disease, the weakness.”* - *“After the surgery, of course, there was no eating and no drinking for 3 months, (…) At that point I was so weak. (At) the end of January 2019, I was so weak that I could barely walk by myself.”* - *“Weakness in general during the treatments? Oh, yes. Yes.”* - *“I don’t have the strength. The chemo has made me more anemic.”* - Moderator: “Do you experience weakness?”   - *“Yes. Yes, absolutely. I cannot… I can’t open things. I can’t put pressure… My arms are not nearly as strong, and that’s another reason why I’m trying to walk. I’m trying to build up my strength a little bit because I am so… I get so frustrated because I can’t do something. I can’t open something. I can’t tighten something. I can’t pick something up. It’s annoying.”* - Moderator: “You also mentioned some walking problems because you were feeling weak. For the past few weeks do you think that there has been a change in your experience with these signs/symptoms?”   - *“Oh, those are signs/symptoms – the weakness and all that stuff – it’s stuff that was happening in 2018 and the beginning of 2019. By mid-2019 I start to recover and progressively got part of my strength back. I had to rebuild the body completely basically, so I started to work out, and I started to play tennis, and keep doing more and more and more.”* - *“I grew up on a farm doing tons of physical work. And I’ve really tried to upkeep a degree of strength. And so some of this is physical weakness.”* - *“I love snowboarding in the winter. I haven’t been able to do that because I just don’t think I… I don’t have the strength right now because I’m… I’ve been kind of skin and bones for the last year and a half. I would say it’s definitely changed my life.”* |
| Diarrhea | - *“Back when I was on the FOLFOX. I just had everything going on. I had constipation. I had diarrhea. I had these eating issues. Everything was happening.”* - *“I was on a chemo drug called irinotecan. The nickname for is it ‘I Run to the Can’ because it causes such severe diarrhea, and that really got me. And diarrhea, dehydration, fatigue.”* - *“Oh, it’s a 10. You can’t go anywhere. I have to stay by the toilet.” “Not so much. It normally happens in the morning, and I deal with it. I would call it maybe 3 out of 10, 2 out of 10.”* |
| Vomiting | - *“With conventional chemotherapy, obviously, nausea and vomiting are big on the list (…) When you feel that your body wants to get rid of that [acid]. So that creates the feeling of nausea. Again, for me, it usually results in vomiting to get rid of some of that acid and stomach contents and then things can settle down.”* - *“And this [vomiting] is after the surgery, and it’s in part because I have a much smaller stomach and a shortened esophagus, and I don’t have a valve between them.”* - *“Because I think it was the tumor in my stomach that was making me sick… to vomit and not be hungry.”* - *“Certain odors and smells can throw you off, or you can be very sensitive to those things.”* - *“I started vomiting out of the blue. I would just be sitting and then I’d vomit and then I’d feel fine… It was… emotionally, it was scary the first time, but I started experiencing more vomiting, I started to lose weight, I felt more weak. And I would say that lasted… When I first started chemo, I just started vomiting a lot, every day, sometimes twice a day. Every time I ate, I would vomit.”* - *“He [doctor] said this was going to be a heavy and harsh treatment, and that’s what it’s been doing. Every day I vomit like 10 to 15 times a day.”* - *“Not vomiting in the traditional way. Vomiting would be caused by the food (getting) stuck… not because you have stomach sick because there’s no stomach anymore.”* - *“Well, I can’t vomit anymore. Without a stomach you don’t throw up right. I call it gagging.”* - Moderator: “How bothersome is it for you?”   - *“10.”* - *“Well, when I successfully vomit, I’m very pleased. Before that I’m not at all. Before the vomit it’s 10 out of 10.”* |
| Early satiety | - *“That [early satiety] is ongoing because of my lack of stomach.”* - *“Oh, yeah. Yeah. Yeah, I get that, but I wouldn’t relate it to the treatments. I relate that to the no stomach.”* - *“That (early satiety) was mainly before diagnosis that I reported that.”* - *“I started experiencing early satiety during meals as well as ultimately, I started experiencing pain after meals. That led me to consult my primary care physician.”* - *“It’s normal for me now to feel full quickly, because again, I don’t have a stomach. So, I just eat meals that are appropriate to what I can handle now. So, I don’t have anything like I had before, where it was an issue. I just have to eat smaller meals.” “I had possibly vague signs/symptoms as a little bit of sometimes fullness, difficulty swallowing, and specifically, I had noticed a little weight loss.”* |
| Difficulty in swallowing | - “*No, not now. It [difficulty swallowing] was during the radiation.”* - Moderator: “Difficulty swallowing?”   - *“Not having a stomach. Yeah, that’s definitely not having a stomach.”* - *“The stent is a helpful thing. The stent is just a… it’s a mechanical tube that they’ve inserted inside the esophagus that holds it open and pushes back the inflamed tumor and allows an opening to pass food through.”* - *“Yes, I started to in the months leading up to the diagnosis, and that… It’s not surprising because you had a golf ball-sized tumor right at the bottom of your esophagus.”* - *“It’s not in the same sense as it was before. Before you have difficulty swallowing because you have a tumor in the tube.”* - *“I had possibly vague signs/symptoms as a little bit of sometimes fullness, difficulty swallowing, and specifically, I had noticed a little weight loss that I had not attempted to lose weight, and I had lost about 10 pounds. In August of 2010, I saw a gastroenterologist and had an EGD (esophagogastroduodenoscopy) performed. That was how I got started.” “Currently the swallowing probably, but if you’d asked me 4 days ago when I had acute gas pains, I would certainly say that those bothered me the most.”* |
| Taste alterations | - *“The minute I got off of that drug, the oxaliplatin, it was fine. You’ll notice that anybody that complains about it tastes disgusting or nothing tastes good, that drug makes it happen.”* - *“I’m less… the chemo has reduced my sensitivity to taste certain food.”* - *“I had to go for the only foods that were attractive to me, and those foods were things like very simple bland foods like potatoes. I ate a lot of hash browns during that summer (…) food tastes which, again, contributes to the problem of eating and holding things down. (…) I would have this metallic taste in my mouth.”* - *“When I'm finished pumping the chemo in, that day that I usually have half a day and the next day are the worst days of… and then it can linger on a little into the next day. I get a metallic taste in my mouth, and so things don’t taste as good but I force myself to eat. I get a little more nauseous.” “I was never an Alfredo person, but now that tastes okay to me. So more creamier dishes… my tastes have changed a lot. Things just don’t taste… Spices are too much for me, they just taste… Everything tastes… like 10 times more powerful, I guess.”* |
| Abdominal pain | - *“That’s not having a stomach. That’s not having a stomach because of the way things affect me now.”* - *“It’s usually there after I eat, for about an hour or so after I eat.”* - *“Not really abdominal pain, except for… I haven’t had this in a while, but I would say with my first two treatments maybe, especially my second treatment. (…) And it was all related to having a bowel movement, I think.”* - *“Very painful. It was like a stabbing pain (…) constant pain that it made it difficult to do anything.”* - *“Abdominal, I mean, it’s here and there, so it’s not too crazy. The liver is the one that’s like… even sometimes it’s like somebody is kicking me in the liver. That’s how much it hurts. (..) A lot of liver pain that I’m seeing right now, also lower abdominal pain.”* - *“Very painful. It was like a stabbing pain in the sort of what I know now is where my liver is located. (…) The pain was really intense, really, really severe. It was a solid 10.”* - *“I’m going to say it [stomach pain] was a 20 the night I dialed 911.” “I’m glad you mentioned that because this is something that I’ve had periodic trouble with since the surgery, and I think it’s because of trapped pockets of gas. And at its worst, we’re talking 9.”* |
| Pain | - *“Thank God. It’s like throbbing. I’ll get a throbbing (pain).”* - *“When I went on chemo, or right when my cancer started, it burns sometimes. Well, fairly often, actually.” “All my long-term side effects seem to be related to surgery. (…) I have some back pain right where that surgery was. Again, on a scale of 0 to 10, today, it's no more than a 2.”* |
| Weight loss | - *“I probably just with chemo I probably lost 10 pounds in the last month, but it hadn’t really disturbed me yet.”* - *“I would say more to the disease than the treatment.”* - *“That’s both... I think the chemo was really what killed me with the weight loss.”* - *“I lost 25 pounds... I would say more to the disease than the treatment, but the treatment doesn’t help because it does make me more nauseous (…) Lining of my stomach is very thick, so I just don’t have room for a lot of food... I can only take a few bites. Otherwise, I’ll throw up.”* - *“I had noticed a little weight loss that I had not attempted to lose weight, and I had lost about 10 pounds. In August of 2010, I saw a gastroenterologist and had an EGD performed. That was how I got started.”* - *“I started experiencing unexplained weight loss. I started experiencing early satiety during meals as well as, ultimately, I started experiencing pain after meals. That led me to consult my primary care physician.”* - *“When I lost a lot of weight during the summer of 2019 when I was on the first treatment, I really was having issues with my appearance. I was really skinny. I was having difficulty walking.”* - *“I lost almost 90 pounds from my normal weight. So, I lost all my muscle and a lot of fat, of course. I just have no strength and no stamina. I fall asleep a lot.”* - *“I have lost more weight primarily due to nausea and lack of appetite.”* - Moderator: “How much does that bother you on a scale of 0 to 10?”   - *“A lot because… a lot because I keep losing weight.* - Moderator: “What number would you give it?”   - *“10.”* - Moderator: “The weight loss itself, is this something that bothers you?”   - *“Yeah. Again, I lost 75 pounds. I lost about 30% of my body weight. Yeah. That was distressing. I felt like I was melting away.”* - *“I did lose weight but I had some weight to lose.”* |
| Neuropathy | - *“I have peripheral neuropathy in my feet, which is caused by treatment. It was caused by the different platins. I’m so used to that that it’s like second nature now.”* - *“No. Not currently. Just in the past. It’s related to a category of chemotherapy drug called platinum chemo, like oxaliplatin, which is very typically given as part of a protocol for stomach cancer. That can cause neuropathy, and tingling, and everything you are describing.”* - *“That one [neuropathy] definitely was the treatment.”* - *“I have neuropathy in my feet from the FOLFOX… When it first came on that was the reason for stopping the FOLFOX, by the way. The tumors had shrunk dramatically, and the neuropathy was just getting way too intense and painful. I was having it in the hands and feet, but since then it’s gone away in the hands, so I don’t really have it in the hands anymore, and it’s really reduced quite a lot in the feet, but it’s still there.”* - *“Numb, tingling… Tingling is the best word, I guess, I can use. It doesn't interrupt my life. I'm able to... I walk normally and do anything I want to, but it's always there.”* - *“Right now, it’s the fatigue and nausea, and the neuropathy, and the hair loss.” “Most bothersome for me is the neuropathy cold sensitivity. That one is the constant. It never goes away’”* |
| Hair loss | - *“When I started the ENHERTU about a week after my infusion I noticed that my hair (…) I would say I’ve lost, shoot, 80% of my hair currently.”* - *“Yes. It [hair loss] was due to chemo.”* - *“Taxol also causes hair loss, so that was goodbye to the hair.”* - *“Hair? Yes. I lost my entire hair and now it’s just growing back… It was from the treatment. From the trial.”* - *“I lost my hair two times. I lost it in 2011, and I lost it again in 2018 and 2019 (…) That's definitely a side effect associated with one of those big drugs. Taxol, that was a bad one. Just forget your hair. Forget your hair.”* - *“I lost every bit of my hair, hair on my head and my beard, body hair, everything. That all came back except the top of my head.”* - *“I lost all my hair.”* - Moderator: “How would you differen(tiate) between the hair loss and hair thinning?”   - *“Well, hair loss, I consider you lose all your hair. Thinning, it's just kind of like, it seems to be gone in spots. But because I have long hair, it kind of covers it. I don't know how to describe it, but I generally have thick hair and it just feels thinner.”* - Moderator: “Have you reported any hair loss?”   - *“I’ve lost all my hair three times, two or three times, and it’s grown back a little bit. It grows back sort of patchily, slowly, and unevenly now.”* - “*When they first shaved my head, I cried. And my husband got his head shaved, too (…) with me. I just sat in the car and felt bad. (…) I always wear a different hat and they call me the ‘Hat Lady’.”* - *“That was kind of bothersome. I* *think it is to most women, it's kind of, yeah, it is. It's awful. …maybe some people tolerate it better than I did, but I did not like that. I would call that even an 8. That didn't sit well with me.”* - *“Well, I’d say probably I’d say not too much. But I, I'd say it's up there with like 8. Because it just makes me look so different that I'm not used to looking in the mirror and seeing this person. So, I'd say 8.”* |
| Constipation | - *“The last time I got constipation was after the surgery, or after anesthesia you do have a bit of constipation.”* - *“I was able to wean myself off of a lot of the secondary medications that I didn’t need anymore, and antinausea medications like Zofran because that causes constipation. If I take one pill I’m dealing with constipation for a few days. (…) I need to be drinking lots of water, lots of fruits, be eating lots of fibrous foods and that sort of thing.” “I would say both but more to the treatment, and the reason why I say to the disease is because of the fact that I have had to change how I eat. That, I think, affected my bowel movements.”* |
| Flatulence | - *“I’m going to attribute that [gassiness] to the disease.”* - *“I think that’s another sort of little issue that I’ve had since my start of cancer treatments.”* - *“Absolutely. Again, I attribute that to the lack of having a stomach and what food I eat, all that kind of stuff.” “It's probably due to … when I'm doing the medications, but it could also be you’re supposed to stay away from like fresh vegetables and I've always been a vegetable eater, so I will eat salad.”* |
| Loss of appetite | - *“I would say that’s one of those things that’s intertwined with the disease and the treatment because in the months leading up to diagnosis my appetite completely dropped.”* - *“Treatment. It does say the same. It says it gives you low appetite.”* - *“I just don’t feel like eating, or I don’t… Nothing tastes good. It’s like things that I used to love I don’t like the taste of now. I almost feel like the inside of my mouth my tastebuds are burnt. They’re just kind of funky feeling, and that came along with the chemo, (…) so that affects my appetite (…) That’s just from the tastebuds and the taste of things.”* - *“I never have an appetite, since I had most of my stomach removed. I have no appetite. I haven’t reported hunger, ever, since I had most of my stomach removed. Because the vagus… What do you call it? A vagotomy or something like that? The vagus through my stomach was severed.”* - *“Oh, yeah. Well, part of the struggling with the partial gastrectomy, you have to learn your whole re-eating. You have just a little piece of a stomach. (…) Now you’re going to eat smaller. I had to set alarms on my cell phone to remind me to eat because the part of the stomach that was removed produces the hunger. Till today, I still don’t get hungry. I just have to remind myself to eat.”* - *“I want to say general fatigue, but a little lower energy, and I wouldn’t doubt that some of that if not most of it is due to my not full eating potential.”* - *“We went out to a really nice dinner on Derby Day and I was not hungry at all. That was bothersome, so I’d say that was probably an 8. Another time, we were out with family hiking on a weekend trip and I didn’t feel hungry and everybody was having fun eating and everything tasted horrible to me and I just didn’t want to eat. Again, that was probably another 8 because it ruined the fun kind of thing.”* |
| Mouth sores | - Moderator: “Any mouth sores or pain in your mouth?”   - *“Not now, however, back in the FOLFOX the very first treatment a little bit. Yeah, I’d get some little mouth sores here and there.”* - “Moderator: “Any mouth sores?   - *“During some of the early treatments, yes.”* - *“The worst that happens is I get terrible, terrible mouth sores from the Xeloda.”* - *“They (mouth sores) can become very, very, very painful. It’s just hard to imagine that a mouth sore could be that painful, but they are. When they get to the point where I can’t tolerate them, I don’t want to tolerate them, there’s not much that I can do besides take pain medicine. None of the things that had been suggested or tried has really helped that much... They are white-ish. Well, it’s usually one white-ish patch. It starts off red and then gets bigger and bigger. It’s probably no more than a quarter inch in size at its biggest. It’s usually in a place like underneath my tongue, or on the roof of my mouth. I can stop taking the drugs and then the mouth sores will recede.”* - Moderator: “Any mouth sores?” - *“Concentrating like just around my lips and corner of my mouth. I started bleeding a little bit, so it’s not like big welts, but it’s just swollen a little funny, a little purple, and it’s just they’re sensitive, so I just have to be careful.” “That's kind of like a cold sore on your mouth, in your mouth or on your lip. Mostly it's been on my lip area.”* |

aGC/GEJC, advanced/metastatic gastric cancer/gastroesophageal junction cancer.

### Supplementary figures

Supplementary Figure 1 Preliminary conceptual model


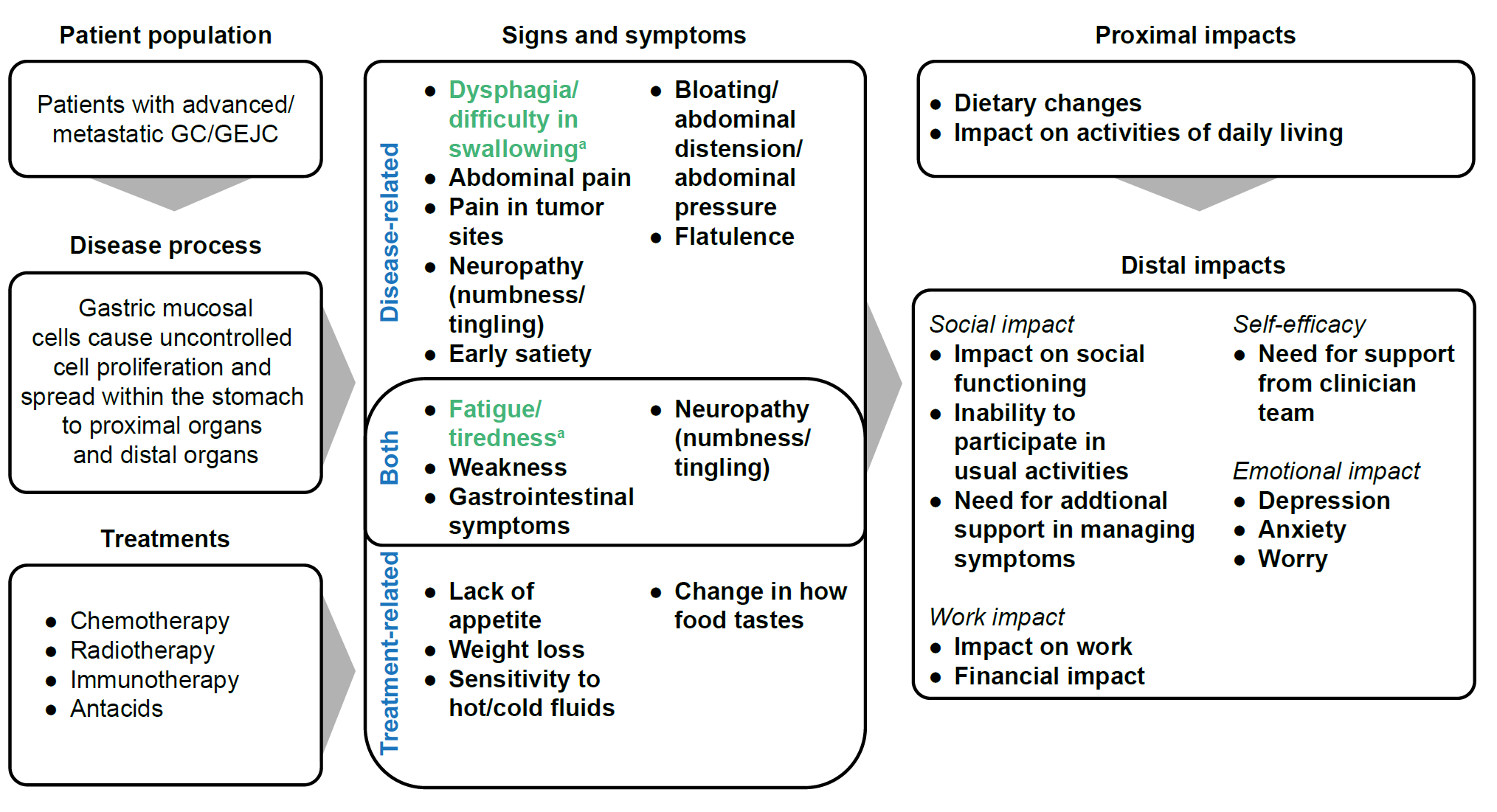
All concepts of high prevalence (at least one literature source that mentions prevalence ≥ 50% spontaneously reported by patients) are prioritized and bolded.

^a^Concepts indicate variation among GC and esophageal cancer.

There were variations in the most commonly reported signs/symptoms between cancers, with fatigue or tiredness the most common symptom reported by participants with GC (GC, n = 20, 80%; esophageal cancer, n = 13, 51%) and dysphagia the most common for esophageal participants (esophageal cancer: n = 66, 65%; GC: n = 8, 32%).

GC, gastric cancer; GC/GEJC, gastric cancer/gastroesophageal junction cancer.
